# Mutant *HVEM* in lymphoma disrupts BTLA and CD160 regulation of anti-tumor signaling

**DOI:** 10.3389/fimmu.2026.1914960

**Published:** 2026-09-11

**Authors:** Wai Wai Lin, Sarah Huang, Olivia Balmert, Ryan Bjordahl, Brian C. Ware, Aref Moshayedi, Jennifer Nguyen, Carl F. Ware, John R. Šedý

**Affiliations:** Laboratory of Molecular Immunology, Sanford Burnham Prebys Medical Discovery Institute, La Jolla, CA, United States

**Keywords:** anti-tumor response, cancer mutation, checkpoint protein, lymphoma, melanoma, phospho-proteomics

## Abstract

**Introduction:**

Somatic mutations can rewire tumor–immune interactions to favor cancer progression. Here, we define the functional impact of missense mutations in Herpesvirus entry mediator (HVEM, TNFRSF14), a receptor frequently altered in lymphoma and melanoma.

**Methods:**

We first assessed binding interactions between cell-expressed HVEM proteins containing mutations identified in human B cell lymphoma and its ligands LIGHT (TNFSF14), B and T lymphocyte attenuator (BTLA), and CD160. We next assessed the capacity for these ligands in a soluble or membrane expressed format to engage these mutant HVEM receptors and activate NF-κB signaling using luciferase assays. We additionally used NK92 cells in cytotoxicity assays with K562 and JVM13 target cells expressing these HVEM mutants to compare wild-type and mutant HVEM costimulation of NK cell lysis. We used proteomic analysis to identify BTLA and CD160 interacting proteins in NK92 and Jurkat cells, and phospho-proteomic analysis to identify signaling pathways activated by these receptors. We used a mouse model of B cell lymhoma expressing ovalbumin (TBL-OVA) and a mouse model of melanoma (B16) to assess how HVEM expression in these tumors regulated their expansion *in vivo*. Additionally, we used OT1 transgenic CD8+ T cells that recognize TBL-OVA cells and were derived from wild-type, Btla-deficient, or Cd160-deficient animals to assess the role of BTLA and CD160 in regulating anti-tumor responses.

**Results:**

We found that HVEM mutations identified in human B cell lymphoma cluster in the extracellular domain. These mutants selectively disrupt BTLA and CD160 binding, and showed weaker activation of NF-κB in response to ligand binding compared to wild-type HVEM. These mutants also showed reduced costimulation of NK92 lytic function compared to wild-type HVEM. Proteomics revealed that BTLA engagement suppresses interferon signaling, whereas CD160 regulates MYC pathway activity. Functionally, HVEM expression and BTLA activation restrained growth of TBL-OVA tumors *in vivo*, while CD160 was required for optimal natural killer (NK) and tumor-specific CD8⁺ T cell responses.

**Discussion:**

Our data illustrate an immuno-evasion strategy used by lymphoma to interrupt HVEM-activated inhibitory signaling in cis, and CD160 costimulatory signaling in trans, demonstrating the pressure to acquire HVEM mutations. The HVEM network thus plays a critical role in regulation of anti-tumor immunity, making it an attractive target for clinical intervention in cancer.

## Graphical Abstract

**Figure:**
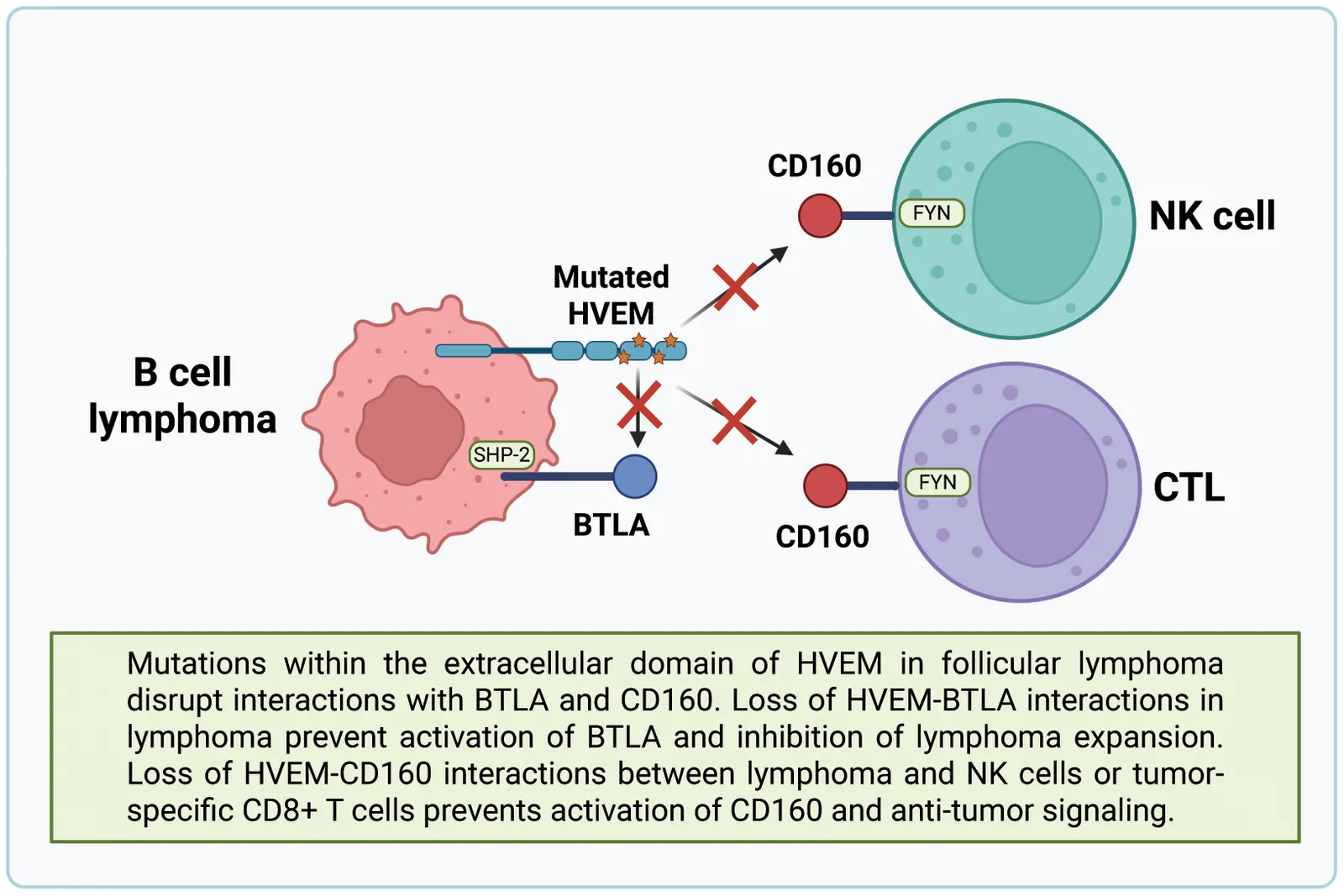

## Introduction

Activation of cytolytic cells is critical in immune responses to cancers, and tumors with mutations in immune recognition pathways are associated with more aggressive tumor outgrowth and mortality (1). In human follicular lymphoma (FL) a common secondary genetic lesion occurs in Herpesvirus entry mediator (HVEM, TNFRSF14) (20% deleted, 18% mutated), and in diffuse large B cell lymphoma (DLBCL) the gene encoding *HVEM* is also a frequent lesion (10-20% mutated) (2–8). A majority of the missense mutations in a subset of lymphoma were concentrated in the extracellular domain and were predicted to impact HVEM binding to its ligands. Additionally, HVEM mutations have been identified in skin, colorectal, lung, liver, and breast cancer (9). HVEM interacts with multiple ligands expressed in the immune system including the tumor necrosis factor (TNF) superfamily cytokines, homologous to lymphotoxin, exhibits inducible expression and competes with HSV glycoprotein D for binding to herpesvirus entry mediator, a receptor expressed on T lymphocytes (LIGHT) and Lymphotoxin-α, and the immunoglobulin superfamily members B and T cell attenuator (BTLA) and CD160 that serve as checkpoint regulators (10). It remains unclear how these mutations in *HVEM* or altered ligand interactions may influence lymphoma fitness within the tumor microenvironment.

The TNF receptor superfamily member HVEM is a focal point for mutation in cancers, infectious disease, and autoimmunity (11–17). In humans, LIGHT, BTLA, and CD160, all activate NF-κB signaling downstream of HVEM following receptor engagement, while HVEM activates the receptors BTLA and CD160 resulting in bi-directional signaling between neighbor cells (18–20). Cells co-expressing HVEM and BTLA or CD160 can also form cell-intrinsic complexes of these proteins that prevent accessibility of these receptors to extracellular ligands due to steric hindrance (21–23). In T cells, BTLA was first shown to engage inhibitory signaling pathways including the activation of SH2-domain-containing protein tyrosine phosphatases (SHP)-1 and 2 (24). Additionally, BTLA has been shown to regulate B cell receptor signaling, toll-like receptor signaling in dendritic cells, and IL-7 signaling in γδ T cells (26–28). However, in human natural killer (NK) cells HVEM can also promote cytolytic and pro-inflammatory pathways through CD160 as a host counter measure to human cytomegalovirus (HCMV) (29). The mechanism of CD160 signaling is unclear, although a transmembrane isoform was shown to recruit the tyrosine kinase LCK and activate NK cells (30). Recent work describing CD160-deficiency in mice confirms its pro-inflammatory function in NK cells (31). In follicular lymphoma both HVEM and BTLA were found to exhibit heterogeneous expression, suggesting the HVEM-BTLA axis directly regulates lymphoma *in cis* (25). In contrast, CD160 was found to be absent in normal B cells and follicular lymphoma, suggesting a potential role for HVEM-CD160 signaling *in trans* (32).

Biologics that block ligand interactions with checkpoint receptors have proven successful in releasing the immune system to treat a variety of cancers (33). Here, we define the biophysical interactions between HVEM network proteins derived from lymphoma and illustrate their impact on HVEM network signaling. We show that several lymphoma HVEM mutants ablate binding to both BTLA and CD160. BTLA signaling was required to regulate lymphoma expansion *in vivo* in a cell-intrinsic manner. Additionally, CD160 in NK cells and CD8^+^ T cells was required for efficient anti-tumor responses. Together, these data indicate a selective evolutionary pressure for the acquisition of somatic *HVEM* mutations in lymphoma that enable both unregulated expansion and immune evasion.

## Results

### Somatic *HVEM* mutations in lymphoma target the HVEM extracellular domain

**Figure 1:**
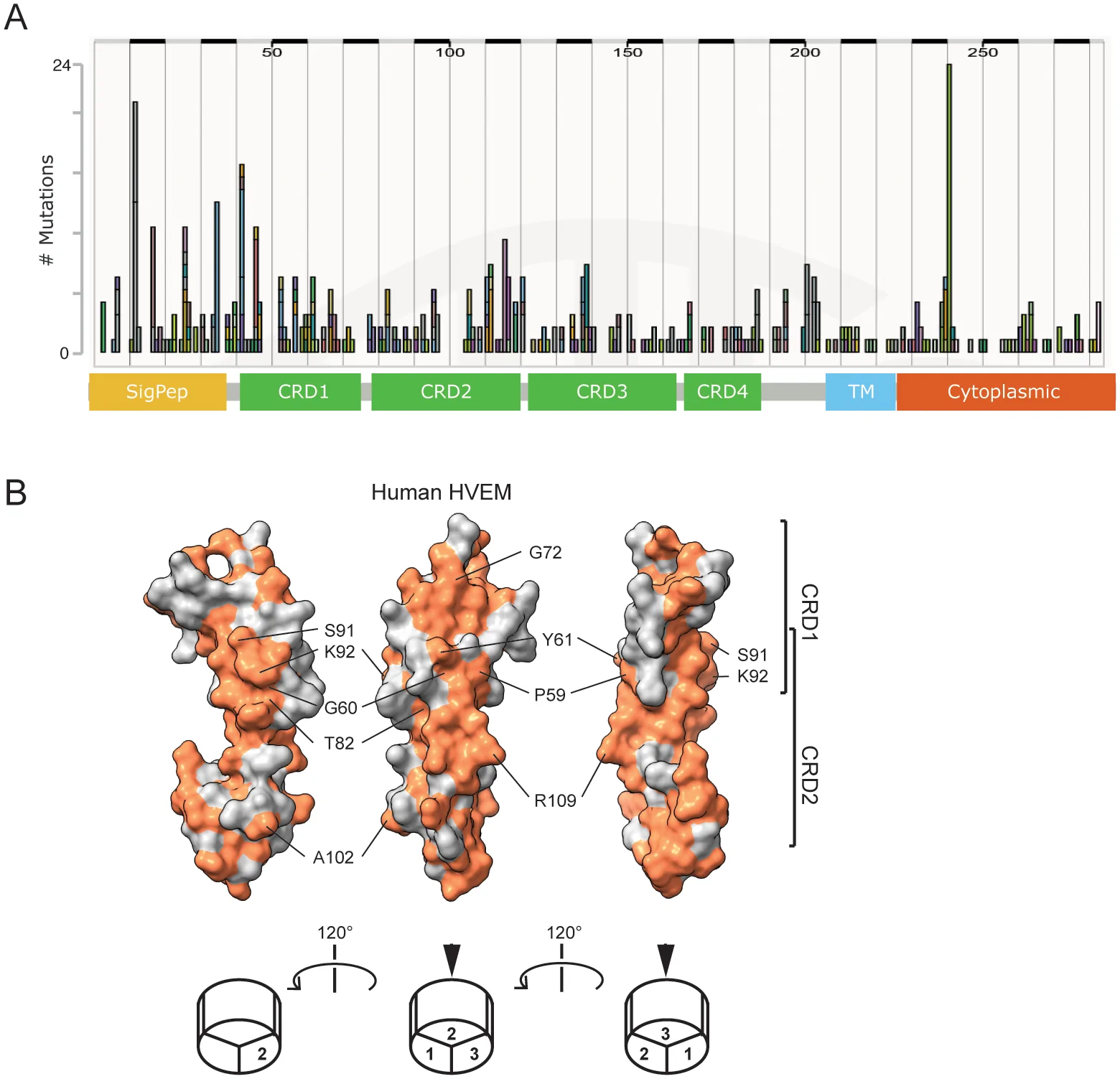
Identification of HVEM-ligand binding mutants in human lymphoma. **(A)** Histogram shows the frequency of mutations identified in *HVEM* from the COSMIC database (Forbes et al., 2017) with a diagram of the HVEM protein domain sequence shown below. **(B)** Images show three structures of human HVEM CRD1 and CRD2 in gray (Protein Data Bank ID code 2AW2) rotated 120° about the y-axis showing mutated surface residues from all human lymphoma mutations colored in coral.

We first cataloged lymphoma mutations in *HVEM* that were available in public datasets (The Cancer Genome Atlas (TCGA), International Cancer Genome Consortium (ICGC), Catalogue of Somatic Mutations in Cancer (COSMIC)) to identify mutational hotspots in the HVEM protein that could indicate mutagenic pressures (34, 35). Among 296 unique non-silent mutations identified across lymphomas, we confirmed that the majority (197/296) are present within the extracellular domain (ECD) of the HVEM protein (**Figure 1**) (2). Additionally, many (79/296) are present within the signal peptide including several that disrupt the start codon. Within the ECD, missense mutations were almost exclusively located within the first three cysteine-rich domains (CRD) (108/121) that is required for ligand binding. Truncating nonsense or frameshift mutations were distributed throughout the HVEM ECD with many mutations predicted to generate soluble ECD proteins.

**Figure 2:**
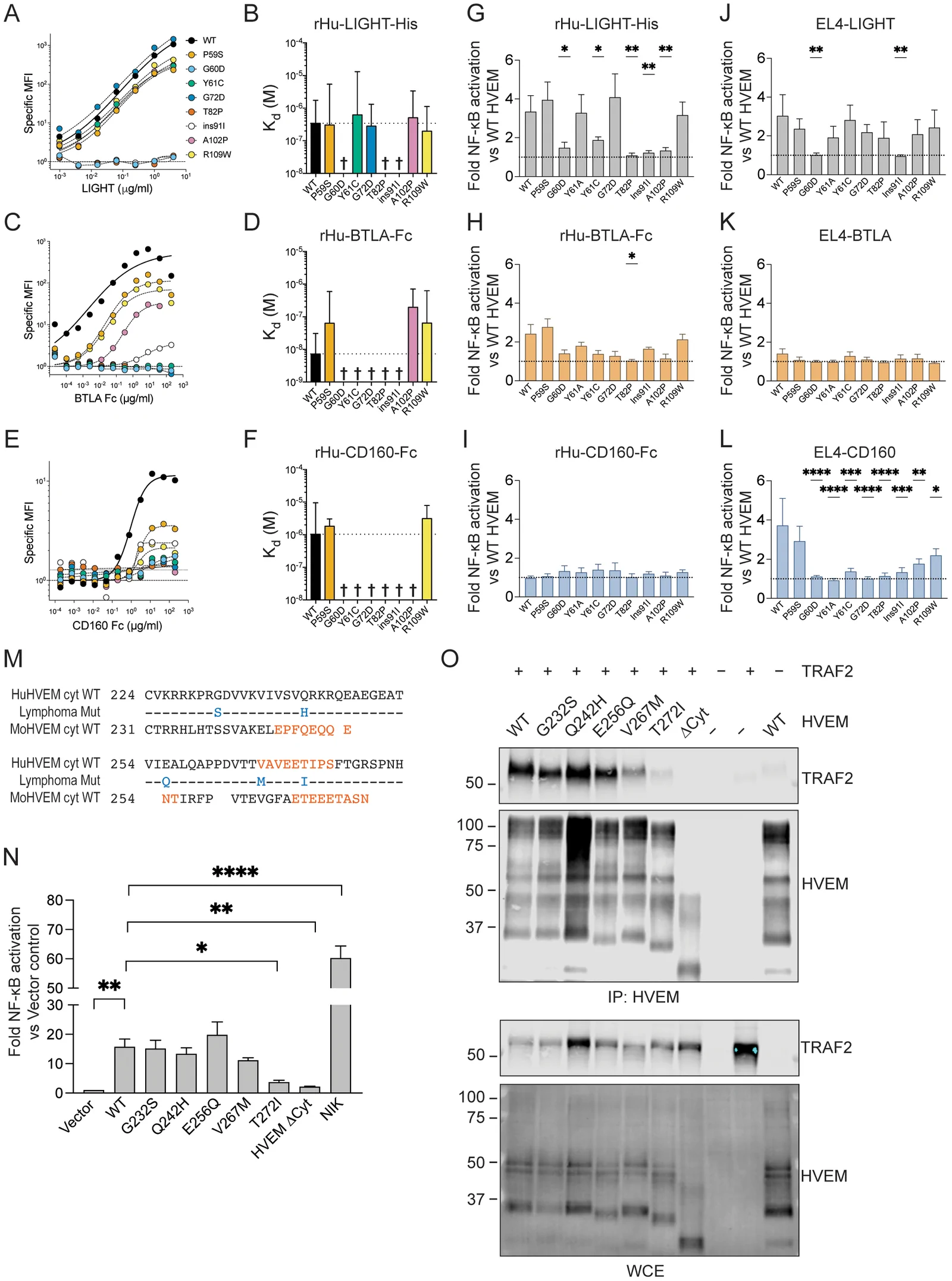
Analysis of alterations in HVEM signaling by lymphoma ECD mutants. **(A–F)** Binding of HVEM ligands to lymphoma mutants were measured in 293T cells transduced with wild-type or mutated human HVEM. Cells were stained with the indicated concentrations of LIGHT-FLAG **(A)**, BTLA-Fc **(C)**, or CD160-Fc **(E)**. Graphs show binding curves for the indicated HVEM mutants from a representative experiment. K_d_ of ligand binding to HVEM mutant proteins were plotted in **(B, D, F)** Geom mean ± SD; at least three experiments per mutation; †, not determined. **(G–L)** Activation of NF-κB by mutant HVEM receptors was measured by luciferase assay in 293T cells transduced with reporters and wild-type or mutant constructs. Cells were activated for 6 hr with 10 μg/ml soluble LIGHT-FLAG **(G)**, BTLA-Fc **(H)**, or CD160-Fc **(I)**, or with EL4 cells expressing membrane LIGHT **(J)**, BTLA **(K)**, or CD160 **(L)**. Graphs show the fold increase in NF-κB activation by HVEM ligands. Data was analyzed together using two-way ANOVA with multiple comparisons to the controls using Fisher’s LSD test. Mean ± SEM; results pooled from at least three experiments per ligand. *p < 0.05; **p < 0.01; ***p < 0.001; ****p < 0.0001. **(M)** Alignment of the human and mouse HVEM cytoplasmic domains illustrating TRAF2 binding motifs (red) and positions of mutations identified in human lymphoma (blue). **(N)** Intrinsic HVEM-induced NF-κB activation in the absence of HVEM ligands was measured by luciferase assay in 293T cells transduced with reporters and wild-type, lymphoma mutant, a cytoplasmic deletion mutation (ΔCyt), or NIK constructs. Cells were activated for 6 hr following transduction. Graph shows the fold increase in NF-κB activation compared to cells transduced with vector control. Data was analyzed by one-way ANOVA with multiple comparisons to the control using the Holm-Šídák test. Mean ± SEM; results pooled from at least three experiments. *p < 0.05; **p < 0.01; ****p < 0.0001. **(O)** Anti-FLAG immunoprecipitation of HVEM-FLAG from 293T cells co-transfected with TRAF2-MYC and HVEM-FLAG containing wild-type or mutant HVEM sequences as indicated (top). ΔCyt, cytoplasmic domain truncation. Protein expression was confirmed by total Western blot (bottom).

We predicted that somatic *HVEM* mutations acquired in lymphomas could interrupt conserved HVEM interactions with its ligands, and subsequent function. We examined several lymphoma-associated mutations and assessed mutant receptor binding to BTLA, CD160, and LIGHT (3–6). In our initial assessment of mutations in the *HVEM* gene we selected for analysis the most frequent mutations at each residue that did not result in a truncation or at cysteine residues that were likely to disrupt the structure of the HVEM extracellular domain. We first verified surface expression of several HVEM missense mutants, and then examined how these mutants impacted ligand binding (**Figures 2A–F**; **Supplementary Figure 1A**). These mutations impacted HVEM interactions in either one of two ways. First, a few mutations disrupted binding to LIGHT, BTLA, and CD160 completely, including G60D, T82P, and Ins91I. Second, most mutations retained binding to LIGHT, but disrupted interactions with both BTLA and CD160 either partially or completely, including P59S, Y61C, G72D, A102P, and R109W. These results are consistent with previous observations by our group and others that HVEM Tyr^61^ is critical for binding both BTLA and CD160 (36–38). We confirmed that these *HVEM* mutations disrupted NF-κB signaling activated by HVEM in response to soluble or membrane bound ligands (**Figures 2G–L**). Activation of NF-κB by soluble LIGHT was disrupted by G60D, Y61C, T82P, Ins91I, and A102P, while activation of NF-κB by membrane LIGHT was disrupted by G60D and Ins91I, mirroring results from LIGHT binding assays (**Figures 2G, J**). Interestingly, we found that soluble BTLA triggered HVEM dependent NF-κB signaling, while membrane BTLA was less effective (**Figures 2H, K**). In contrast, membrane CD160 potently triggered HVEM dependent NF-κB signaling, while soluble CD160 was less effective (**Figures 2I, L**). Activation of NF-κB by soluble BTLA showed a trend for reduced signal for all mutants except P59S and R109W (**Figure 2H**). Membrane CD160 activated a robust NF-κB signal that was reduced by all mutants except P59S (**Figure 2L**). Together, these somatic mutations define a hierarchy of ligand binding with preferential loss of interactions with CD160 and BTLA that is associated with either intact or disrupted LIGHT interactions. Additional HVEM mutations have been described since our initial assessment and these may exhibit more diverse trends for disrupted interactions with LIGHT, BTLA, and CD160. Among the mutations tested, we find that while each of these ligands could activate NF-κB, BTLA may be more active in a soluble form while CD160 is most active in a membrane form. Interestingly, serum levels of the soluble form of BTLA that is potentially derived from alternative splicing is associated with worse cancer prognosis (39).

We next examined whether mutations in *HVEM* were linked to disruption of intrinsic NF-κB signaling due to overexpression of *HVEM*. Among all somatic *HVEM* mutations in lymphoma, 5 missense and 1 truncating mutation are within the cytoplasmic domain sequence including G232S, Q242H, E256Q, V267M, and T272I (**Figure 2M**). Interestingly, HVEM Thr^272^ is within a TRAF2 binding domain which may impact TRAF2 recruitment (40) We observed similar intrinsic NF-κB activity in cells expressing wild-type HVEM and the HVEM mutants G232S, Q242H, E256Q, and V267M (**Figure 2N**). In contrast, the T272I mutation showed a significantly reduced NF-κB signal that was similar to a cytoplasmic deletion HVEM construct. We next determined whether these mutant cytoplasmic domains were capable of recruiting TRAF2 to HVEM (**Figure 2O**). Each of the cytoplasmic mutant constructs was capable of precipitating TRAF2 except for the T272I mutant and HVEM with a cytoplasmic deletion that each showed reduced NF-κB signal (40). Together, these data indicate that the TRAF2 recruitment domain is required for HVEM overexpression-induced NF-κB signaling. Additionally, monoallelic truncation or deletion mutations within the extracellular region of *HVEM* are predicted to decrease ligand induced HVEM signaling. However, it remained unclear whether these mutations were linked to increased lymphoma fitness.

### Cell-intrinsic HVEM restrains lymphoma proliferation *in vivo*

**Figure 3:**
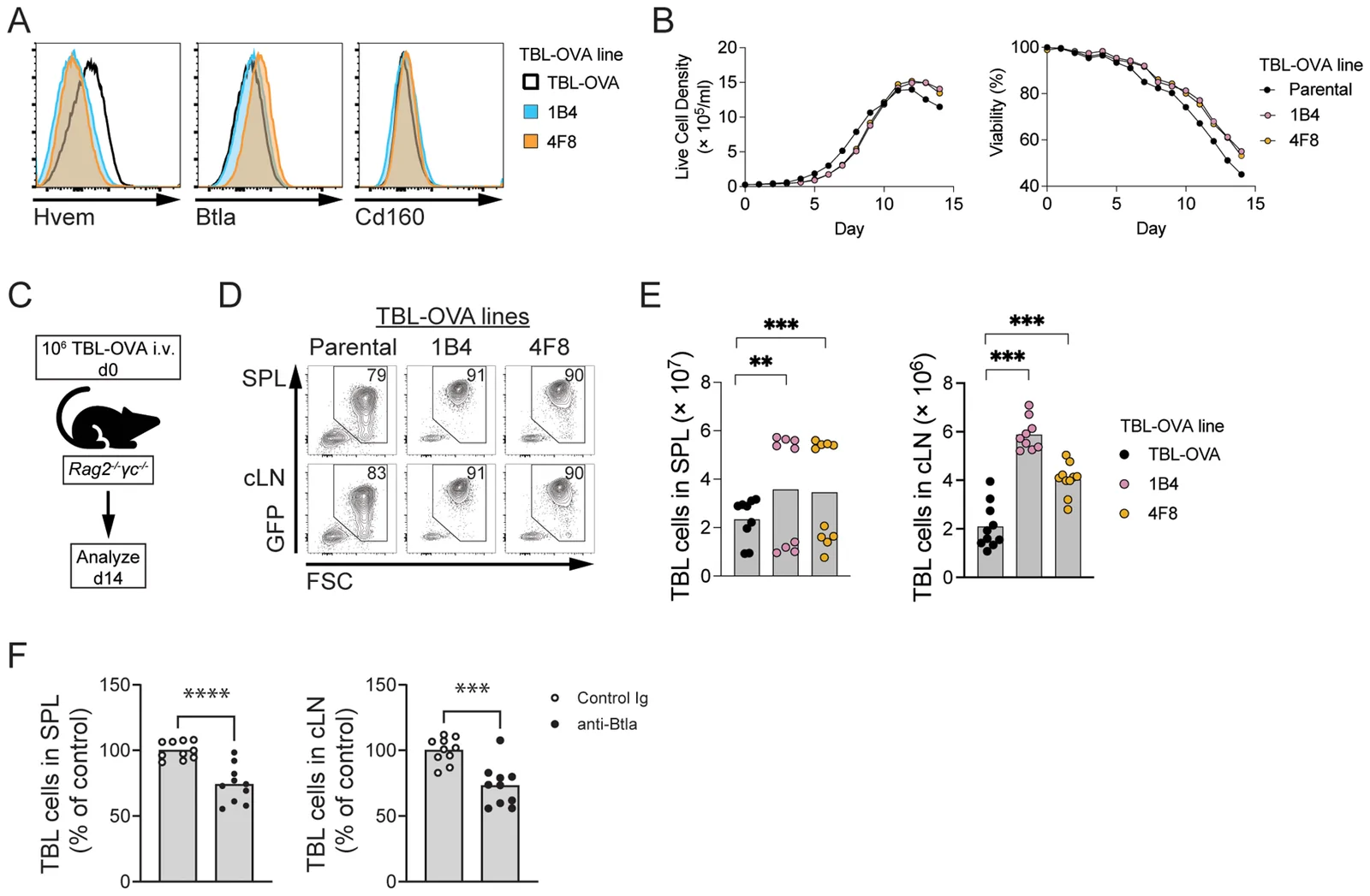
HVEM suppresses lymphoma growth in a cell-intrinsic manner *in vivo.* **(A)** Histograms show expression of mouse Hvem, Btla, and Cd160 in parental TBL-OVA lymphoma cells and Hvem CRISPR KO lines 1B4 and 4F8. **(B)** Graphs show growth curves of TBL-OVA, 1B4, and 4F8 TBL lines *in vitro*. Graphs show cell density (left) and viability (right). Data is representative of n=2 experiments. **(C)** Diagram for inoculation of *Rag2^-/-^Il2rg^-/-^* with TBL cell lines to measure cell expansion in immune deficient animals *in vivo*. **(D, E)** Contour plots show identification of CD19^+^GFP^+^ TBL cells *in vivo* in spleen (top) and cervical lymph node (bottom) in animals inoculated with TBL-OVA, 1B4, or 4F8 cells **(D)**. Graphs show the number of TBL-OVA cells present in the spleen (left) and cervical lymph node (right) at the time of harvest **(E)**. **(F)** Graphs show the cellularity of TBL-OVA cells in animals treated with anti-Btla in the spleen (right) and cervical lymph nodes (right). Data was analyzed using one-way ANOVA with multiple comparisons to the control using the Holm-Šídák test **(E)** or Student’s t test **(F)**. Mean ± SEM; results pooled from at least two experiments each for CRISPR KO lines and antibody treatment. **p < 0.01; ***p < 0.001; ****p < 0.0001.

We next expressed *HVEM* mutants in several B cell lymphoma cell lines to examine their impact on lymphoma growth and function. Wild-type and mutant HVEM proteins did not affect cell-intrinsic growth *in vitro* or expression of receptors associated with B cell maturation including IgM, HLA-DR, CD86, CD40, and CD27 (**Supplementary Figures 2A, B**). In order to test how lymphoma-associated lesions in *HVEM* could specifically impact the makeup of the tumor microenvironment *in vivo*, we adopted an experimental model of B cell lymphoma using adoptive transfer of TBL-OVA cells that show pathologic hallmarks of Burkitt’s lymphoma (41, 42). We first generated TBL-OVA cells with ablated HVEM expression to model *HVEM* mutations in human B cell lymphoma using multiple *Hvem* specific guide RNA and Cas9 protein transfection (1B4 and 4F8) (**Figure 3A**). Each of the TBL-OVA clones showed similar growth curves, indicating HVEM was not necessary for proliferation in culture (**Figure 3B**). Using these cells, we assessed whether HVEM regulated cell-intrinsic growth of lymphoma *in vivo* by transfer to immune-deficient hosts (**Figure 3C**). Interestingly, both *Hvem^-/-^* clones showed increased numbers of cells in the spleen and the cervical lymph node, the latter of which is the major site of TBL engraftment (**Figures 3D, E**). We next examined whether increased tumor burden in the absence of HVEM was due to a lack of BTLA activation in lymphoma through therapeutic administration of anti-BTLA (clone 6A6, hamster Ig) (26). Importantly, this antibody was previously shown to be a non-depleting agonist for BTLA that prevented graft-versus-host disease in an animal model *in vivo* (43, 44). Antibody activation of BTLA *in vivo* resulted in a reduced tumor burden in the spleen and cervical lymph node (**Figure 3F**). Thus, HVEM limits tumor burden in a cell-intrinsic manner *in vivo*, that may in part be due to activation of BTLA inhibitory signaling.

### HVEM ligation of BTLA and CD160 activate distinct signaling pathways

**Figure 4:**
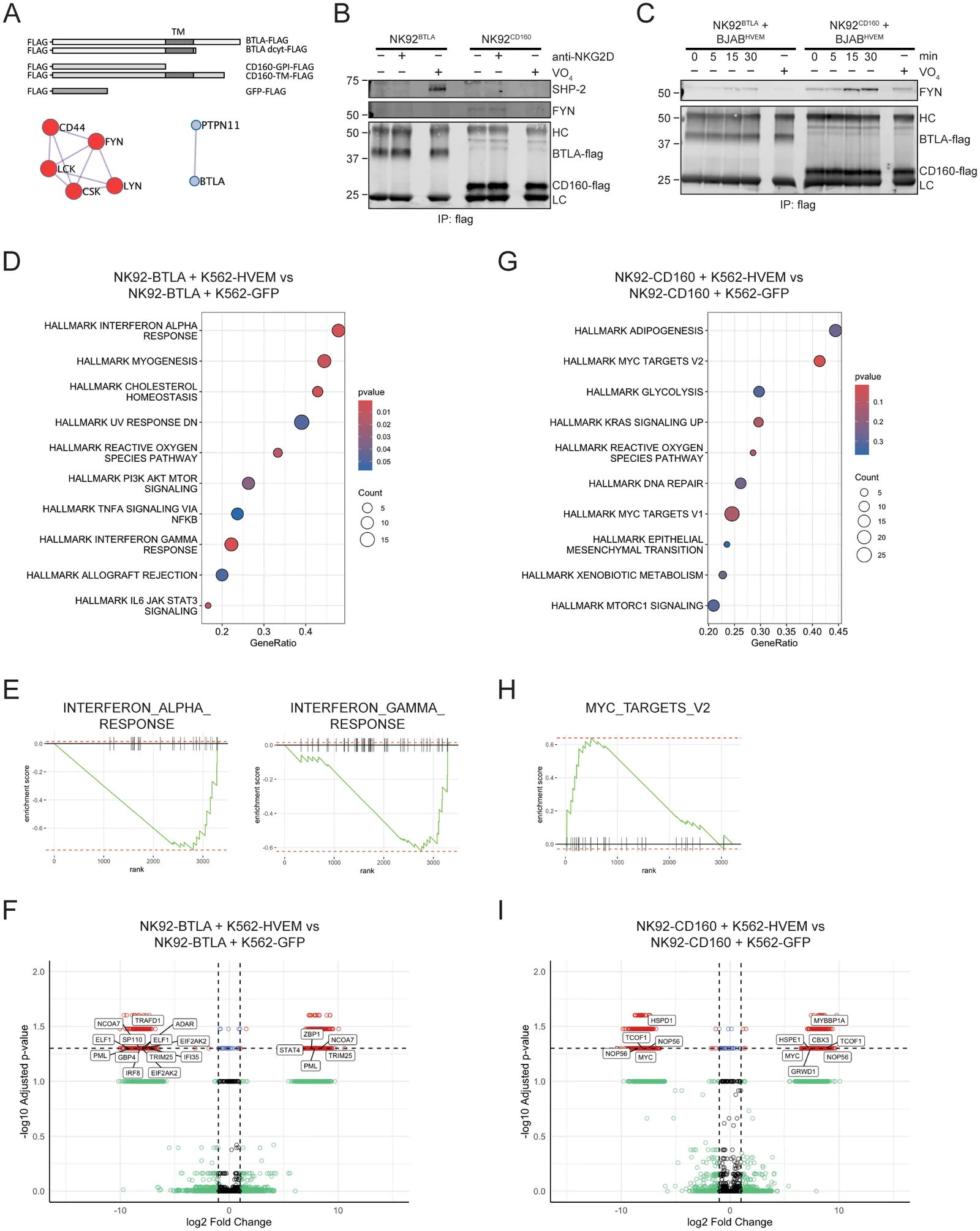
Identification of HVEM activated BTLA and CD160 signaling pathways. **(A)** The constructs used to precipitate BTLA, BTLAΔCyt, CD160-GPI, CD160-TM, and GFP interacting proteins is indicated (top) and the network analysis of proteins identified in regulated in antibody activated Jurkat-CD160 (bottom left) or Jurkat-BTLA (bottom right) generated using Metascape (Zhou et al., 2019). **(B)** Anti-FLAG immunoprecipitation of BTLA-FLAG or CD160-FLAG in NK92 cells expressing these receptors that were stimulated with anti-NKG2D or VO_4_. Blots show VO_4_ induced recruitment of SHP-2 to BTLA and constitutive association of FYN to CD160. HC, heavy chain; LC, light chain. **(C)** Anti-FLAG immunoprecipitation of BTLA-FLAG or CD160-FLAG in NK92 cells stimulated with BJAB cells expressing HVEM. Blots show kinetics of FYN recruitment to BTLA and CD160. **(D–F)** Gene set enrichment analysis of differential phospho-protein activation in NK92-BTLA cells stimulated with K562-HVEM cells compared to K562-GFP cells. Enriched gene sets are plotted in **(D)**, with the INTERFERON_ALPHA_RESPONSE and INTERFERON_GAMMA_RESPONSE plots in **(E)** Volcano plot of differentially phosphorylated proteins is shown in **(F, G–I)** Gene set enrichment analysis of differential phospho-protein activation in NK92-CD160 cells stimulated with K562-HVEM cells compared to K562-GFP cells. Enriched gene sets are plotted in **(G)**, with the MYC_TARGETS_V2 plot in **(H)** Volcano plot of differentially phosphorylated proteins is shown in **(I)** p values were calculated using the *GSEA* package in R.

Lymphoma-associated HVEM mutants may act as a ligand for BTLA and CD160 in neighboring cells within the tumor microenvironment including both lymphoma or tumor infiltrating lymphocytes (TIL). We examined activation of BTLA and CD160 by HVEM to determine how lymphoma may regulate these signaling pathways *in vivo*. We initially used immunoprecipitation mass spectrometry to identify BTLA and CD160 receptor proximal signaling proteins in NK92 cells and Jurkat cells as model lymphocytes. We first confirmed BTLA recruitment of the SHP-2 phosphatase to BTLA (**Figure 4A**). In cells expressing the glycophosphatidylinositol (GPI)-linked form of CD160, we identified several co-precipitating proteins including the tyrosine kinases FYN, LCK, LYN, and CSK that mediate lymphocyte activation, and the CD44 adhesion receptor. Notably, detergent insoluble lipid rafts have been shown to anchor GPI-linked proteins such as CD160 as well as palmitoylated signaling proteins such as FYN, LCK, and LYN, thus providing a potential mechanism for CD160 signal transduction (45). We confirmed induced association of BTLA with SHP-2 in NK92 cells following VO_4_ stimulation (**Figure 4B**) (24). We additionally observed constitutive association of FYN with CD160 that was increased following activation with HVEM-expressing BJAB cells (**Figures 4B, C**).

We further evaluated how CD160 and BTLA activation regulated global protein phosphorylation using NK92 cells transduced with either BTLA or CD160, and stimulated with K562 target cells expressing HVEM. HVEM stimulation of BTLA induced significant changes in 1514 phosphorylation sites across 1032 proteins, including BTLA. Gene set enrichment analysis indicated a negative impact to the Hallmark Interferon Alpha Response (FDR = 0.024) and Hallmark Interferon Gamma Response (FDR = 0.030) pathways (**Figures 4D–F**). Notably, we previously observed a reduction in the Hallmark Interferon Gamma Response in anti-CD3 activated mouse CD4^+^ T cells that were treated with an agonist anti-BTLA antibody (46). HVEM stimulation of CD160 induced significant changes in 1408 phosphorylation sites across 981 proteins, including Fyn-binding protein (FYB) that is an adapter for FYN, which regulates IL-2 expression. Although no pathways reached significance, the Hallmark Myc Targets V2 pathway showed the strongest enrichment (NES = 1.56, nominal p = 0.0098) (**Figures 4G–I**).

We performed a similar phospho-proteomic analysis of Jurkat cells expressing either BTLA or CD160 and stimulated with the agonist antibody anti-BTLA (clone MIH26) or anti-CD160 (clone 688327) respectively. In BTLA-expressing Jurkat cells the pathways showing strongest enrichment were for Hallmark Interferon Alpha Response (NES = -1.69, nominal p = 0.022) and Hallmark IL2 STAT5 Signaling (NES = -1.56, nominal p = 0.039) (**Supplementary Figures 3A–C**). In CD160-expressing Jurkat cells the pathway showing strongest enrichment was for Hallmark Myc Targets V1 (NES = -1.41, nominal p = 0.0083) (**Supplementary Figures 3D–F**). While none of the enriched pathways in Jurkat cells reached significance following multiple testing correction, this was not unexpected due to the small fold changes observed in proteomic datasets. However, the trend for BTLA to regulate interferon responses and for CD160 to regulate MYC signaling is consistent with the data from NK92 cells, indicating unique and distinct functions of BTLA and CD160 that are conserved between T cells and NK cells.

### HVEM stimulates NK cell anti-tumor responses through CD160

**Figure 5:**
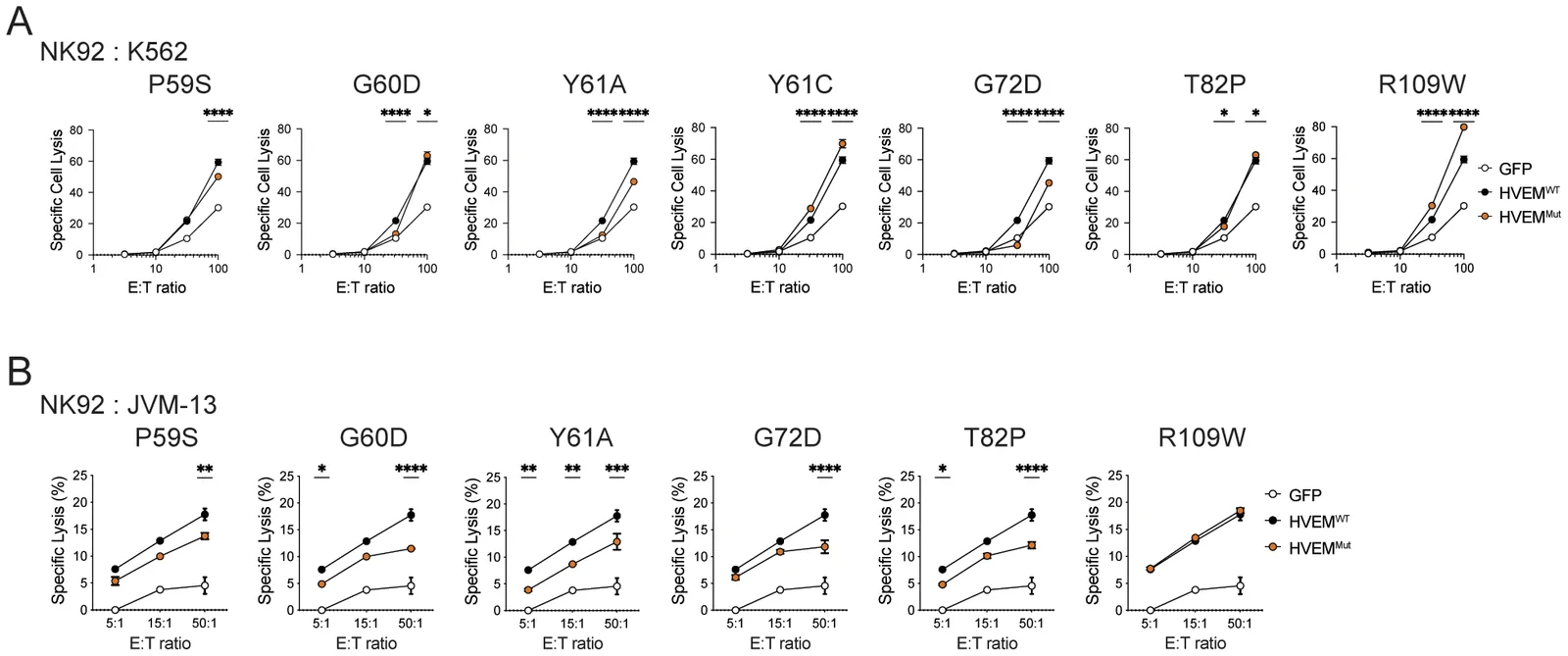
Lymphoma ECD mutants reduce cytolytic activation. **(A, B)** NK92 cells were incubated at indicated effector to target (E:T) ratios with K562 cells **(A)** or JVM13 cells **(B)** stably transfected with wild-type or mutant HVEM or control vector in cytolysis assays. Graphs indicate the specific lysis of different ECD mutants compared to wild-type HVEM and control vector following incubation with effector cells. Data for all mutants was analyzed by two-way ANOVA with multiple comparisons to the control using the Holm-Šídák test. Mean ± SEM, representative of at least two experiments. *p < 0.05; **p < 0.01; ***p < 0.001; ****p < 0.0001.

In human lymphoma, ECD mutations in HVEM may prevent activation of CD160 and BTLA, resulting in altered lymphocyte activation. To this end, we examined how wild-type or ECD mutant HVEM could affect NK anti-tumor cytotoxicity *in vitro* using NK92 cells as a surrogate for primary NK cells to induce lysis of K562 leukemia cells. We previously demonstrated that both primary NK cell and NK92 cell lysis of K562 cells could be costimulated with HVEM proteins (29). The mutations Y61A and G72D mutants showed the greatest reduction of cytotoxicity compared to wild-type HVEM, consistent with their specific impact in disrupting HVEM activation of CD160 (**Figures 2L**, **5A**). To confirm the role of HVEM in costimulating NK cell lytic activity we next examined NK92 responses to several human B cell lymphomas expressing wild-type or mutant HVEM. Among several B cell lymphoma lines, we found that the JVM13 cell line induced weak killing by NK92 cells, but that HVEM expression rendered these cells more sensitive to NK cell killing compared to controls (**Figure 5B**; **Supplementary Figure 4A**). Notably, JVM13 cells expressing the lymphoma-associated HVEM mutants P59S, G60D, Y61A, G72D, and T82P were all lysed less efficiently than JVM13 cells expressing wild-type HVEM. In contrast, the HVEM R109W mutant did not impact NK92 lysis of JVM13 cells. While the R109W HVEM mutant showed reduced affinity to BTLA and CD160, this reduction was modest compared to other mutants, and it did not impact cell-expressed CD160 activation of HVEM signaling to the same extent as other mutants (**Figure 2**). Together these data suggest the weaker interaction between the R109W mutant with CD160 was sufficient to activate NK92 cell lytic activity to the same extent as wild-type HVEM. Interestingly, the HVEM mutants showed a more pronounced reduction in NK cell lysis of the JVM13 cells compared to the K562 cells. Activation of NK cell cytotoxic function is threshold gated, determined by the net positive signal by activating and inhibitory receptors and is influenced by ligand affinity, density, and duration of signal (47). Interestingly, JVM13 cells express high levels of BTLA that may compete with NK cell-expressed CD160 (**Supplementary Figure 2B**). Thus, the impact of reduced affinity of the HVEM mutants to both CD160 and BTLA may result in greater reduction in lysis of BTLA-expressing cells.

We next determined whether activation of BTLA or CD160 activation could modulate NK92 cell cytolytic activity using agonist anti-BTLA (MIH26) or anti-CD160. Antibody activation of BTLA showed a trend for reduced NK92 lysis of JVM13 cells, consistent with our previous findings that HVEM activation of BTLA reduced NK92 cell cytolytic function (**Supplementary Figure 4D**) (29). We confirmed that the anti-CD160 clone 688327 binds cell expressed CD160 and that it competes with HVEM for CD160 binding (**Supplementary Figures 4B, C**). Anti-CD160 enhanced NK92 lysis of JVM13 cells, indicating that this antibody may function as an agonist similar to HVEM (**Supplementary Figure 4E**). While the mechanism of NK92 cell lysis of JVM13 cells is unknown, NK92 cell lysis of K562 cells was previously shown to require expression of LFA-1 and NKG2D (48). Together, these data support a role for NK cell-expressed BTLA in regulating NK cell lysis of lymphoma, and a role for CD160 in potentiating NK cell lysis of lymphoma cells. Importantly, the mutations in HVEM that disrupt CD160 binding also prevent efficient activation of NK cell cytotoxic function.

**Figure 6:**
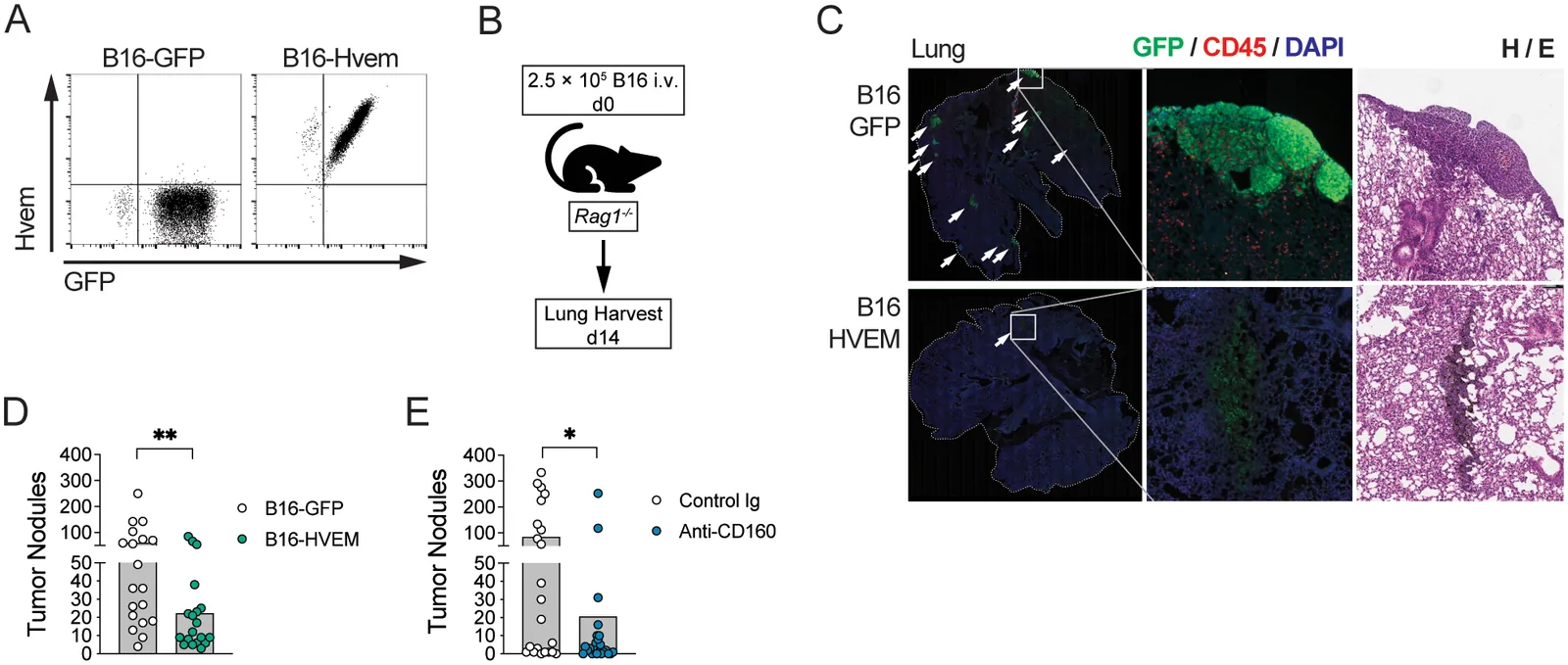
Tumor expressed HVEM promotes *in vivo* clearance through CD160. **(A)** Retrovirus transduced B16-F10 tumor cells were stained with anti-HVEM to confirm expression. Dot plots show GFP-sorted control cells (left) or mouse HVEM expressing cells (right). **(B)** Diagram of experiment for *in vivo* analysis of NK cell anti-tumor response using B16 lung nodule metastasis model. **(C)** Lungs were harvested from *Rag1^-/-^* animals 14 days after i.v. inoculation with 2.5 × 10^5^ B16 tumors. Histology images show the presence of tumors (arrows) in the lungs of animals with B16-GFP inoculation (top row) and the in the lungs of animals with B16-HVEM inoculation (bottom row). **(D, E)** Graphs show numbers of tumors within lungs at time of harvest in animals inoculated with B16-GFP compared to B16-HVEM melanoma **(D)**, and in animals treated with anti-CD160 (clone CNX46-3) or isotype control on day 0 and day 2 during inoculation with B16-GFP melanoma **(E)**. Data was analyzed using Student’s t test. *p < 0.05; **p < 0.01.

In order to confirm whether the HVEM-CD160 intereaction regulated anti-tumor responses by NK cells *in vivo*, we used the well-defined B16 melanoma model in which mouse NK cells are specifically activated by these tumors. As previously observed, CD160 is highly expressed in human and mouse NK cells compared to other cells and express the lowest levels of BTLA (**Supplementary Figures 5A-D**). Of note, *HVEM* is a target of microphthalmia-associated transcription factor in melanocytes, and *HVEM* is highly expressed in both melanoma and lymphoma (**Supplementary Figure 5E**) (9, 49). We examined melanoma progression in a lung metastasis model using systemic inoculation of NK cell-sensitive B16 melanoma transduced with *Hvem* cDNA (mouse HVEM) (**Figures 6A, B**). Interestingly, animals receiving *Hvem*-expressing B16 cells developed fewer and smaller lung tumor nodules than animals receiving control B16 cells (**Figures 6C, D**). Reduced formation of B16-HVEM tumor nodules was dependent on host expression of *Il2rg* that is required for NK cell development, and NK cell depletion resulted in unrestricted tumor engraftment (**Supplementary Figures 5D–F**), confirming a requirement for NK cells in clearance of B16 melanoma. We next evaluated whether direct activation of CD160 was required for NK cell clearance of melanoma cells. We confirmed that the agonist anti-CD160 clone CNX46–3 did not block HVEM binding to CD160, allowing co-binding of these receptors and maintaining HVEM activation of CD160 (**Supplementary Figure 6**). Animals treated with this antibody showed reduced lung tumor nodules, indicating that CD160 activation may costimulate NK cell function *in vivo* (**Figure 6E**). Together, these data show that melanoma-expressed HVEM limits tumor burden in the presence of CD160-expressing NK cells, indicating that the HVEM-CD160 interaction activates NK anti-tumor activity.

### HVEM activates CD8^+^ anti-lymphoma responses *in vivo*

The makeup of infiltrating immune cells within diverse B cell lymphoma subtypes includes a variety of tumor specific adaptive and innate cytotoxic effectors, as well as cells contributing to germinal center reactions (8, 9, 13). As a first approximation of microenvironment interactions within lymphoma tissues harboring genetic alterations in *HVEM*, we evaluated immune signatures from expression profiles associated with the British Columbia Cancer Agency (BCCA) validation cohort used to confirm FL genetic signatures and associated clinical outcomes in biopsy specimens identified in the GLSG2000 trial (**Supplementary Figure 7A**) (50, 51). Interestingly, lymphomas harboring *HVEM* missense mutations in HVEM showed reduced CD8^+^ T cell and resting dendritic cell signatures (**Supplementary Figures 7B, C**). In lymphoma containing genomic loss of *HVEM*, we additionally observed a reduced signatures for myeloid lineage cells (**Supplementary Figure 7D**). The majority of the mutations in the BCCA validation cohort were localized to the ECD (11/15, 73%), including three that we characterized (G60D, Y61C, T82P) and two (G72V, G72S) that overlapped with the G72D mutation. A recent analysis of the follicular lymphoma tumor microenvironment revealed the presence of both cytotoxic CD8+ T cells and CD4+ T cells (52). Interestingly, the authors noted a reduced antigen presentation signature in tumors harboring mutations in *EZH2*, that overlap with *HVEM* mutations. Together, these transcriptional signature changes within lymphoma suggest that genetic modifications of *HVEM* directly influence cytotoxic T cell function and antigen presentation which ultimately may contribute to tumor selection.

**Figure 7:**
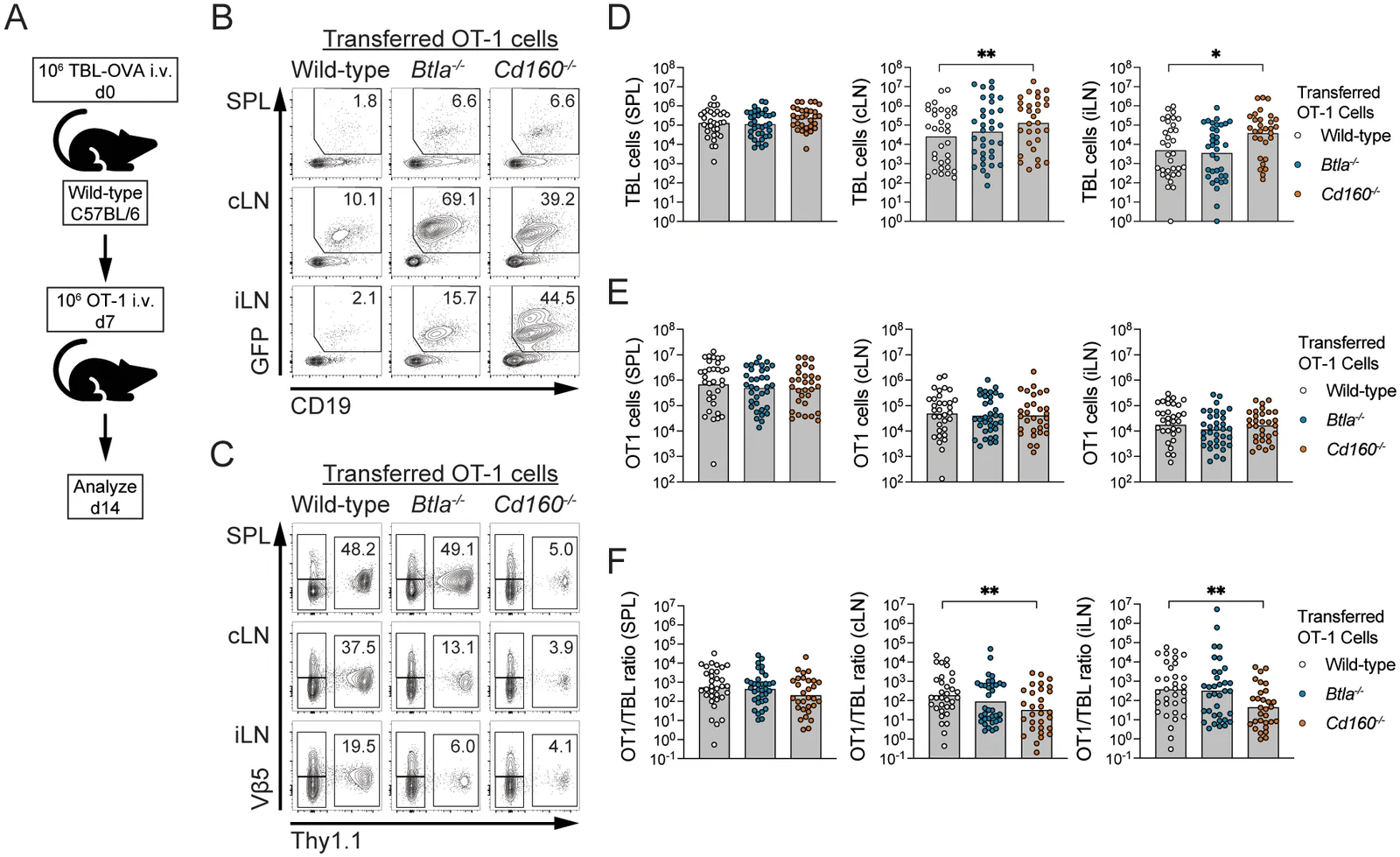
CD160 in T cells is required for efficient anti-lymphoma response *in vivo.* **(A)** Diagram of experiment for analysis of OT1 anti-lymphoma response in TBL inoculated animals. **(B, C)** Contour plots show identification of CD19^+^GFP^+^ TBL cells **(B)** and of CD8^+^Thy1.1^+^Vβ5^+^ T cells **(C)** *in vivo* in spleen (top), cervical lymph node (middle), and inguinal lymph nodes (bottom) in animals inoculated with TBL-OVA cells followed by adoptive transfer of wild-type, *Btla^-/-^*, or *Cd160^-/-^* OT1 CD8^+^ T cells. **(D–F)** Graphs show the number of TBL-OVA cells **(D)**, the number of OT1 cells **(E)**, and the ratio of OT1 cells to TBL-OVA cells **(F)** present in the spleen (left), cervical lymph node (middle), and inguinal lymph node (right) at the time of harvest. Data was analyzed using the linear modeling function in R *p < 0.05; **p < 0.01.

We then used the previously described TBL-OVA B cell lymphoma model to examine the activation of tumor-reactive lymphocytes *in vivo* and whether anti-tumor responses were regulated by expression of BTLA or CD160 receptors. The TBL-OVA lymphoma cell line expresses the OVA antigen that is recognized by OT1 transgenic CD8^+^ T cells. Using OT1 TCR transgenic animals that were bred with *Btla^-/-^* or *Cd160^-/-^* mice, we purified CD8^+^ T cells and transferred these into animals that had been previously inoculated with TBL-OVA lymphoma cells (**Figure 7A**). Notably, in animals that did not receive donor OT1 CD8^+^ T cells we observed an inverse correlation between the frequency of lymphoma cells and CD8^+^ T cells (**Supplementary Figures 7F, G**). Interestingly, in animals receiving *CD160^-/-^* donor OT1 cells we observed increased numbers of tumors in the cervical and inguinal lymph nodes and a trend for increased tumors in the spleen compared to animals receiving wild-type OT1 cells (**Figures 7B, C**). We also observed a trend for decreased numbers of *CD160^-/-^* OT1 cells, and a more significant reduction in the ratio of *CD160^-/-^* OT1 cells to TBL cells compared to animals receiving wild-type cells (**Figures 7D–F**). These results did not appear to be due to changes in total organ cellularity (**Supplementary Figure 7G**). In animals receiving *Btla^-/-^* OT1 cells we did not observe significant changes in tumor burden. Thus, in lymphoma-reactive CD8^+^ T cells CD160 is required for efficient control of tumor numbers *in vivo*. Together these data demonstrate how HVEM interactions with its ligands shapes the tumor microenvironment and how deletions in HVEM can alter the lymphoma burden.

## Discussion

We report a strategy used by lymphoma to interrupt HVEM-activated BTLA inhibitor signaling *in cis*, and CD160 costimulatory signaling *in trans*. We characterize several mutant HVEM proteins arising in lymphoma that lack binding to BTLA and CD160 yet retain interactions with LIGHT. We found that BTLA agonists inhibit inflammatory signaling in the NK92 and Jurkat model cell lines. Additionally, we show that CD160 is associated with the SRC family kinase FYN and potentiates MYC associated signaling pathways. Lymphoma lacking the HVEM protein showed increased expansion *in vivo*, while anti-BTLA reduced lymphoma growth, indicating cell-intrinsic BTLA inhibitory signaling. In contrast, CD160 expression in T cells was required to limit lymphoma expansion. Together, these data demonstrate the pressure to acquire mutant *HVEM* in cancer that prevents activation of both BTLA and CD160, and that these mutations can provide a selective advantage for tumors.

We confirmed the role of BTLA as an immune checkpoint inhibitor regulating T and B cell receptor signaling (20, 27, 53, 54). The mechanism of inhibitory signaling in lymphocytes downstream of BTLA is thought to include activation of SHP-1 or 2, but may involve additional pathways (53, 55). Interestingly, a recent study confirmed high levels of SHP-2 recruitment to BTLA and preferential recruitment of SHP-1 to BTLA with prolonged activation by pervanadate (56). The inhibitory effect of BTLA agonists on early events in antigen receptor signaling is consistent with previous studies demonstrating that CD3ζ and Syk are direct targets of BTLA activity in T and B cells (27, 54). We and others previously found that BTLA stimulation regulates signaling pathways associated with metabolic activation and inflammatory responses (46, 57). Our phosphoproteomic data highlights early signaling events that are consistent with these transcriptional analyses. It was previously shown that deletions of both HVEM and BTLA arise in lymphoma and that lymphoma lacking HVEM exhibit cell-autonomous proliferation (25). In contrast, it was noted that in FL and DLBCL high expression of HVEM and low expression of BTLA were associated with worse prognosis (58). Additionally, BTLA-expression in T cells was found to restrain T cell responses to pre-malignant B cells, indicating a cell-extrinsic role for BTLA in regulating B cell lymphomagenesis (59). Continued investigation is warranted to determine how HVEM contributes to lymphoma fitness in the tumor microenvironment through ligand selection.

Our data show that CD160 is required for efficient anti-tumor signaling both for NK cells responding to melanoma and in CD8^+^ T cells specific for lymphoma. The role of CD160 *in vivo* was previously studied using a cross-species reactive antibody (clone CL1-R2) that was shown to bind endothelial cells within tumor vasculature and to limit tumor engraftment in target tissues (60). However, it was not clear whether NK cells were specifically activated in these models or how CD160 may interact with tumor-expressed ligands. We and others showed that CD160 is required for efficient IFN-γ production by NK cells, which could be activated by HVEM in dendritic cells or macrophages (29, 31). CD160 has been reported to promote both activating and inhibitory signals depending on the cellular context (61, 62). Interestingly, a recent study showed that the GPI-linked receptor CD48 that localizes to lipid rafts can associate with PD-1 in T cells and that CD48 activates PD-1 inhibitory signaling through a SRC kinase-dependent mechanism (63). It is reasonable to suggest that CD160 may also activate inhibitory signaling depending on which proteins are present within CD160-associated lipid microdomains.

A number of efforts have been made to manipulate the HVEM signaling network to treat disease (64). Development of CD160-targeting therapeutics remains challenging due to difficulty in assigning a costimulatory or inhibitory role for this protein. However, the defined role of BTLA as an immune checkpoint has led to efforts to generate BTLA antagonists for cancer therapy. Promising results have recently been reported for the BTLA antagonist tifcemalimab in lymphoma, lung cancer, and esophageal squamous cell carcinoma both as a monotherapy and as a dual therapy with anti-PD-1 (65–67). Interestingly, patients with higher expression of HVEM showed increased responses to therapy. Thus, determination of HVEM network protein expression may indicate clinical responsiveness. In summary, the HVEM network plays a critical role in regulation of anti-tumor immunity and thus is an attractive target for clinical intervention. While development of therapeutics targeting this network is in early stages, initial results suggest that these therapeutics will prove to be valuable tools in the oncologist’s arsenal against cancer.

## Materials and methods

### Cells and surface protein expression

The EL4, 293T, B16, BJAB, RAJI, JVM13, WIL2S, Jurkat, and K562 cell lines were obtained from the American Type Culture Collection (ATCC). The BJAB cell line was obtained from the Deutsche Sammlung von Mikroorganismmen and Zellkulturen (DSMZ).

The TBL-OVA cell line was a gift from Yosef Refaeli, National Jewish Medical and Research Center and was derived following IACUC approval (42) EL4, 293T, and B16 cells were maintained in DMEM with 10% heat-inactivated FBS, antibiotics, L-glutamine and 50 μM 2-ME. BJAB, RAJI, JVM13, WIL2S, Jurkat, K562, -OVA cells and their derivatives were maintained in RPMI with 10% heat-inactivated FBS, antibiotics, L-glutamine, and 50 μM 2-ME. NK92 cells were maintained in RPMI with 8% heat-inactivated FBS and 8% heat-inactivated equine serum, antibiotics, L-glutamine, and 50 μM 2-ME. EL4, Jurkat, NK92, K562, BJAB, JVM13 and B16 cells transduced with retroviruses containing BTLA, CD160, LIGHT, and HVEM expression constructs or control HVEM mutants were produced by round-the-world PCR mutagenesis (**Supplementary Table 1**). Pseudotyped single infection retrovirus was produced by co-transfection of retroviral plasmid, pCG VSVg envelope protein, and Hit60 gag-pol as previously described (20).

### CRISPR knockdown of *Hvem* in TBL-OVA cells

A panel of gRNA were identified to target Hvem for knockdown using the Synthego Design Tool (Synthego) (**Supplementary Table 1**). TBL-OVA cells were transfected with individual gRNA and Cas9 protein and immediately placed in culture. 14 days after culture cells were screened for Hvem expression at which time clones 1B4 and 4F8 were identified as showing successful knockdown.

### Antibodies and Fc fusion protein production

Anti-human HVEM was from BD Biosciences (San Diego, CA). Anti-human BTLA antibody clone MIH26 was obtained from eBioscience (San Diego, CA). Anti-human CD160 was obtained from R & D Systems (Minneapolis, MN) and anti-mouse CD160 was obtained from eBioscience (San Diego, CA). Depleting anti-NK1.1 (clone PK136) was from Bio X Cell (West Lebanon, NH). Donkey anti-human Fcγ F(ab’)_2_ was from Jackson Immunoresearch (West Grove, PA). The Fc fusion proteins were designed and produced as follows: For human BTLA residues 1–150 including the signal sequence were fused at the 3’ end to the human IgG1 Fc regions. For human CD160 residues 1–144 including the signal sequence were fused at the 3’ end to the human IgG1 Fc regions. The Fc fusion proteins were produced in transfected 293T cells grown in CellGro serum free medium and purified by affinity chromatography on protein A columns. Control human IgG1 protein was from Sigma-Aldrich (Saint Louis, MO). Human LIGHT was purchased from R&D Systems.

### Binding assays

Flow cytometric binding assays were performed as previously described (29). Briefly, cells transduced with the indicated HVEM mutants were incubated with Fc ligands for 30 min on ice in buffer (PBS with 2% FBS), washed twice, incubated with anti-Fc secondary for 15 min on ice in buffer, washed twice, and analyzed for ligand binding using flow cytometry. Specific mean fluorescence intensity (MFI) was calculated by subtracting experimental cellular MFI from the cellular MFI of control transduced cells. Binding curves were analyzed using the one site – specific binding module within GraphPad Prism to determine K_d_.

### Immunprecipitation analysis and proteomics

NK92 and Jurkat cells transduced with FLAG-tagged BTLA or CD160 were used to precipitate associated proteins following stimulation. For antibody activation of NKG2D, anti-NKG2D was incubated with NK92 cells at 37 °C for 15 minutes. For VO_4_ treatment, 10 mM VO_4_ was added to cells on ice and cells were incubated at 37 °C for 5 minutes. For HVEM activation of BTLA and CD160, BJAB cells expressing HVEM were incubated with NK92 cells expressing BTLA or CD160 at a 1:10 ratio on ice, and cell mixtures were incubated at 37 °C for 0, 5, 15, or 30 minutes. For antibody activation of BTLA and CD160, anti-BTLA (clone MIH26) or anti-CD160 (clone 688327) were incubated with Jurkat cells expressing BTLA or CD160 for 0, 5, 15, or 30 minutes. For protein ID immunoprecipitation and phosphoproteomics studies, cells were quenched with ice cold PBS and cell pellets were frozen for subsequent batch lysis and mass spectrometry. Samples were processed and analyzed at the Sanford Burnham Prebys Proteomics Core using the Orbitrap Elite mass analyzer (Thermo Fisher). For HVEM-TRAF2 association studies 293T cells were transfected with FLAG-tagged HVEM cytoplasmic domain mutants and TRAF2. For immunoprecipitation studies, stimulated cells were quenched with ice cold PBS and lysed in RIPA buffer at 4 °C for 20 minutes followed by centrifugation at 14,000 rpm, 4 °C. Lysates were incubated with M2 anti-FLAG beads overnight at 4 °C with agitation followed by washing in lysis buffer and boiling in SDS loading buffer containing 1% β-mercaptoethanol for 5 minutes and resolved by SDS-PAGE on 10% Bis-Tris gels (Bio-Rad, Hercules, CA). For whole cell extracts, lysates were boiled in SDS loading buffer and resolved on gels. Proteins were transferred using tank method to PVDF membrane, blocked with LI-COR blocking buffer, and blotted with antibodies against FLAG, TRAF2, FYN, SHP-2, phospho-ERK1/2, total AKT (Cell Signaling), followed by anti-rabbit and anti-goat LI-COR secondary antibodies and visualized using the Odyssey Imager (LI-COR Biosciences).

### Luciferase assays

293T cells were plated at 100,000 cells per well in a 48 well plate, then transfected the following day with pNFkB firefly luciferase DNA to detect specific NF-κB activity, with pRLTK *Renilla* luciferase DNA to control for transfection efficiency, and constructs for expression of HVEM cytoplasmic domain mutants at 4ng/mL. 293T cells expressing mutated HVEM receptors were stimulated the following day with soluble LIGHT, BTLA-Fc, or CD160-Fc proteins, or with EL4 cells expressing BTLA, CD160, and LIGHT at a 1:1 ratio for 24 hours. Stimulated cells were washed in ice cold PBS and lysed in Passive Lysis Buffer (Promega) at room temperature for 15 minutes. Cell lysates were diluted 1:10 in Passive Lysis Buffer then assayed using the Dual-Luciferase Reporter Assay System (Promega) according to manufacturer’s instructions. Briefly, Lucifese Assay Reagent II was added to lysates to activate firefly luciferase activity, followed by addition of Stop & Glo®® Reagent to quench firefly luciferase and activate *Renilla* luciferase activity. Luciferase activity was analyzed using a CLARIOstar Plus plate reader (BMG Labtech). The firefly luciferase signal was divided with the *Renilla* luciferase signal to obtain the relative luciferase signal.

### Cytotoxicity assays

NK92 cytolytic function was assayed first using the CellTox Green Cytotoxicity Assay (Promega). Briefly, K562 were plated at 1,000 cells per well in opaque walled 96 well plates, then incubated with NK92 cells at various E:T ratios (100:1, 31:1, 10:1, and 3.1:1) for 2 hours prior to assay with CellTox Green dye. Cell death was analyzed using the CLARIOstar Plus microplate reader (BMG Labtech). NK92 cytolytic function was repeated using a flow-based assay as previously described (29). Briefly, lymphoma target cell lines and derivatives were labeled with Cell Proliferation Dye e450 (eBioscience) according to the manufacturer’s instructions and cultured with NK92 cells at various E:T ratios for 3 hr prior to labelling with LIVE/DEAD^®^ Fixable Near-IR Dead Cell Stain (Thermo Fisher Scientific, Waltham, MA) and fixation with 2% paraformaldehyde in PBS at 37 °C for 20 min. Labelled and fixed cells were washed in buffer (PBS with 2% FBS) and analyzed.

### Proliferation assays *in vitro*

TBL-OVA cell lines and derivatives were cultured at 25,000/ml. Cells were allowed to grow *in vitro* for 2 weeks without resupplementation of media. Live cell counts and viability were measured every day following initial cell plating. Cell density was calculated as the cell number divided by the culture volume.

### Experimental models of melanoma and pulmonary melanoma metastasis model, and lymphoma

All animal handling and procedures were approved by the Sanford Burnham Prebys Medical Discovery Institute IACUC. For tumor inoculation animals were anesthetized using inhaled isofluorane with 5% induction and 2% maintenance. Animals were euthanized using 30%-70% CO_2_ asphixiation following anesthetization. Lungs from *Rag1^-/-^* or *Rag2^-/-^γc^-/-^* animals were harvested 14 days after inoculation with B16-F0 cells and tumor nodules were counted as described (68). For NK depletion experiments animals were treated with anti-NK1.1 on day 0 and day 2 of tumor inoculation. Perfused lungs were fixed in Fekete’s solution and embedded in paraffin. Serial sections were stained with hematoxylin and eosin to analyze tissue architecture or anti-GFP and anti-CD45 to identify tumors and infiltrating lymphocytes. Whole mount histology sections were scanned using ScanScope^®^ AT2, imaged with Aperio eSlide Manager (Leica Biosystems, Buffalo Grove, IL), and further analyzed with ImageJ software v1.50i (69). Cervical lymph nodes, inguinal lymph nodes and spleens were harvested from *Rag1^-/-^* or *Rag2^-/-^γc^-/-^* animals after inoculation with TBL-OVA cells and homogenized for flow cytometry analysis.

### Lymphoma biopsy immune signature analysis

Gene expression data curated from FL (GSE66166) (51), DLBCL (GSE1138) (70), or Burkitt’s Lymphoma (71) were analyzed with CIBERSORT using the LM22 leukocyte signature matrix (50). FL immune signatures were further stratified according to *HVEM* gene status and DLBCL stratified as germinal center- (GCB), activated- (ABC), or primary mediastinal- (PMBL) B cell lymphoma. Null *HVEM* is defined as either a frame shift insertion/deletion, nonsense mutation, splice site mutation, or translational start site mutation.

### Statistical analysis

HVEM ligand binding curves, K_d_ values, and standard error were calculated from specific MFI using One site – Specific binding module in GraphPad Prism 9. Unless otherwise indicated, in each set of experiments data from all biological replicates were analyzed together for significance using the linear modeling function in R v4.0.0 where each replicate was included as a distinct term in the formula (*response* ~ *replicate* + *term*). Data was plotted using Graphpad Prism 9.

## Data Availability

The datasets presented in this study can be found in online repositories. The names of the repository/repositories and accession number(s) can be found in the article/**Supplementary Material**.

## References

[1] Vesely MD Kershaw MH Schreiber RD Smyth MJ . Natural innate and adaptive immunity to cancer. Annu Rev Immunol. (2011) 29:235–71. doi: 10.1146/annurev-immunol-031210-101324 21219185

[2] Bjordahl RL Steidl C Gascoyne RD Ware CF . Lymphotoxin network pathways shape the tumor microenvironment. Curr Opin Immunol. (2013) 25:222–9. doi: 10.1016/j.coi.2013.01.001 23339845 PMC3646954

[3] Cheung KJJ Johnson NA Affleck JG Severson T Steidl C Ben-Neriah S . Acquired TNFRSF14 mutations in follicular lymphoma are associated with worse prognosis. Cancer Res. (2010) 70:9166–74. doi: 10.1158/0008-5472.CAN-10-2460 20884631

[4] Launay E Pangault C Bertrand P Jardin F Lamy T Tilly H . High rate of TNFRSF14 gene alterations related to 1p36 region in de novo follicular lymphoma and impact on prognosis. Leukemia. (2012) 26:559–62. doi: 10.1038/leu.2011.266 21941365

[5] Lohr JG Stojanov P Lawrence MS Auclair D Chapuy B Sougnez C . Discovery and prioritization of somatic mutations in diffuse large B-cell lymphoma (DLBCL) by whole-exome sequencing. Proc Natl Acad Sci USA. (2012) 109:3879–84. doi: 10.1073/pnas.1121343109 22343534 PMC3309757

[6] Martin-Guerrero I Salaverria I Burkhardt B Szczepanowski M Baudis M Bens S . Recurrent loss of heterozygosity in 1p36 associated with TNFRSF14 mutations in IRF4 translocation negative pediatric follicular lymphomas. Haematologica. (2013) 98:1237–41. doi: 10.3324/haematol.2012.073916 23445872 PMC3729904

[7] Morin RD Mendez-Lago M Mungall AJ Goya R Mungall KL Corbett RD . Frequent mutation of histone-modifying genes in non-Hodgkin lymphoma. Nature. (2011) 476:298–303. doi: 10.1038/nature10351 21796119 PMC3210554

[8] Scott DW Gascoyne RD . The tumour microenvironment in B cell lymphomas. Nat Rev Cancer. (2014) 14:517–34. doi: 10.1038/nrc3774 25008267

[9] Šedý JR Ramezani-Rad P . HVEM network signaling in cancer. Adv Cancer Res. (2019) 142:145–86. doi: 10.1016/bs.acr.2019.01.004 30885361

[10] Ware CF Sedý JR . TNF Superfamily Networks: bidirectional and interference pathways of the herpesvirus entry mediator (TNFSF14). Curr Opin Immunol. (2011) 23:627–31. doi: 10.1016/j.coi.2011.08.008 21920726 PMC3222271

[11] Alcami A . Viral mimicry of cytokines, chemokines and their receptors. Nat Rev Immunol. (2003) 3:36–50. doi: 10.1038/nri980 12511874

[12] Burn Aschner C Loh LN Galen B Delwel I Jangra RK Garforth SJ . HVEM signaling promotes protective antibody-dependent cellular cytotoxicity (ADCC) vaccine responses to herpes simplex viruses. Sci Immunol. (2020) 5:eaax2454. doi: 10.1126/sciimmunol.aax2454 32817296 PMC7673108

[13] Basso K Dalla-Favera R . Germinal centres and B cell lymphomagenesis. Nat Rev Immunol. (2015) 15:172–84. doi: 10.1038/nri3814 25712152

[14] Callahan MK Wolchok JD Allison JP Sharma P . Immune co-signaling to treat cancer. In: Cancer Immunotherapy. Springer New York, New York, NY (2013). p. 211–80. doi: 10.1007/978-1-4614-4732-0_8

[15] Murphy TL Murphy KM . Slow down and survive: Enigmatic immunoregulation by BTLA and HVEM. Annu Rev Immunol. (2010) 28:389–411. doi: 10.1146/annurev-immunol-030409-101202 20307212

[16] Pardoll DM . The blockade of immune checkpoints in cancer immunotherapy. Nat Rev Cancer. (2012) 12:252–64. doi: 10.1038/nrc3239 22437870 PMC4856023

[17] Šedý J Bekiaris V Ware CF . Tumor necrosis factor superfamily in innate immunity and inflammation. Cold Spring Harb Perspect Biol. (2014) 7:a016279. doi: 10.1101/cshperspect.a016279 25524549 PMC4382740

[18] Cai G Anumanthan A Brown JA Greenfield EA Zhu B Freeman GJ . CD160 inhibits activation of human CD4+ T cells through interaction with herpesvirus entry mediator. Nat Immunol. (2008) 9:176–85. doi: 10.1038/ni1554 18193050

[19] Cheung TC Steinberg MW Oborne LM Macauley MG Fukuyama S Sanjo H . Unconventional ligand activation of herpesvirus entry mediator signals cell survival. Proc Natl Acad Sci USA. (2009) 106:6244–9. doi: 10.1073/pnas.0902115106 19332782 PMC2669392

[20] Sedy JR Gavrieli M Potter KG Hurchla MA Lindsley RC Hildner K . B and T lymphocyte attenuator regulates T cell activation through interaction with herpesvirus entry mediator. Nat Immunol. (2005) 6:90–8. doi: 10.1038/ni1144 15568026

[21] Atwell S Cheung TC Conner EM Ho C Huang J Harryman EL . Quantitative detection of the HVEM-BTLA checkpoint receptor cis-complex in human lymphocytes. J Immunol. (2025) 214:565–72. doi: 10.1093/jimmun/vkae057 40073094 PMC12213244

[22] Cheung TC Oborne LM Steinberg MW Macauley MG Fukuyama S Sanjo H . T cell intrinsic heterodimeric complexes between HVEM and BTLA determine receptivity to the surrounding microenvironment. J Immunol. (2009) 183:7286–96. doi: 10.4049/jimmunol.0902490 19915044 PMC2865256

[23] Šedý JR Balmert MO Ware BC Smith W Nemčovičova I Norris PS . A herpesvirus entry mediator mutein with selective agonist action for the inhibitory receptor B and T lymphocyte attenuator. J Biol Chem. (2017) 292:21060–70. doi: 10.1074/jbc.M117.813295 29061848 PMC5743079

[24] Watanabe N Gavrieli M Sedy JR Yang J Fallarino F Loftin SK . BTLA is a lymphocyte inhibitory receptor with similarities to CTLA-4 and PD-1. Nat Immunol. (2003) 4:670–9. doi: 10.1038/ni944 12796776

[25] Bekiaris V Šedý JR Macauley MG Rhode-Kurnow A Ware CF . The inhibitory receptor BTLA controls γδ T cell homeostasis and inflammatory responses. Immunity. (2013) 39:1082–94. doi: 10.1016/j.immuni.2013.10.017 24315996 PMC3909738

[26] Kobayashi Y Iwata A Suzuki K Suto A Kawashima S Saito Y . B and T lymphocyte attenuator inhibits LPS-induced endotoxic shock by suppressing Toll-like receptor 4 signaling in innate immune cells. Proc Natl Acad Sci USA. (2013) 110:5121–6. doi: 10.1073/pnas.1222093110 23479601 PMC3612598

[27] Vendel AC Calemine-Fenaux J Izrael-Tomasevic A Chauhan V Arnott D Eaton DL . B and T lymphocyte attenuator regulates B cell receptor signaling by targeting Syk and BLNK. J Immunol. (2009) 182:1509–17. doi: 10.4049/jimmunol.182.3.1509 19155498

[28] Šedý JR Bjordahl RL Bekiaris V Macauley MG Ware BC Norris PS . CD160 activation by herpesvirus entry mediator augments inflammatory cytokine production and cytolytic function by NK cells. J Immunol. (2013) 191:828–36. doi: 10.4049/jimmunol.1300894 23761635 PMC3702646

[29] Giustiniani J Bensussan A Marie-Cardine A . Identification and characterization of a transmembrane isoform of CD160 (CD160-TM), a unique activating receptor selectively expressed upon human NK cell activation. J Immunol. (2009) 182:63–71. doi: 10.4049/jimmunol.182.1.63 19109136 PMC3472403

[30] Tu TC Brown NK Kim TJ Wroblewska J Yang X Guo X . CD160 is essential for NK-mediated IFN-γ production. J Exp Med. (2015) 212:415–29. doi: 10.1084/jem.20131601 25711213 PMC4354368

[31] Boice M Salloum D Mourcin F Sanghvi V Amin R Oricchio E . Loss of the HVEM tumor suppressor in lymphoma and restoration by modified CAR-T cells. Cell. (2016) 167:405–418.e13. doi: 10.1016/j.cell.2016.08.032 27693350 PMC5221752

[32] Farren TW Giustiniani J Liu FT Tsitsikas DA Macey MG Cavenagh JD . Differential and tumor-specific expression of CD160 in B-cell Malignancies. Blood. (2011) 118:2174–83. doi: 10.1182/blood-2011-02-334326 21715317 PMC5292591

[33] Sharma P Allison JP . The future of immune checkpoint therapy. Science. (2015) 348:56–61. doi: 10.1126/science.aaa8172 25838373

[34] Forbes SA Beare D Boutselakis H Bamford S Bindal N Tate J . COSMIC: somatic cancer genetics at high-resolution. Nucleic Acids Res. (2017) 45:D777–83. doi: 10.1093/nar/gkw1121 27899578 PMC5210583

[35] Zhang J Baran J Cros A Guberman JM Haider S Hsu J . International Cancer Genome Consortium Data Portal--a one-stop shop for cancer genomics data. Database (Oxford). (2011) 2011:bar026. doi: 10.1093/database/bar026 21930502 PMC3263593

[36] Cheung TC Humphreys IR Potter KG Norris PS Shumway HM Tran BR . Evolutionarily divergent herpesviruses modulate T cell activation by targeting the herpesvirus entry mediator cosignaling pathway. Proc Natl Acad Sci USA. (2005) 102:13218–23. doi: 10.1073/pnas.0506172102 16131544 PMC1201609

[37] Compaan DM Gonzalez LC Tom I Loyet KM Eaton D Hymowitz SG . Attenuating lymphocyte activity: the crystal structure of the BTLA-HVEM complex. J Biol Chem. (2005) 280:39553–61. doi: 10.1074/jbc.M507629200 16169851

[38] Kojima R Kajikawa M Shiroishi M Kuroki K Maenaka K . Molecular basis for herpesvirus entry mediator recognition by the human immune inhibitory receptor CD160 and its relationship to the cosignaling molecules BTLA and LIGHT. J Mol Biol. (2011) 413:762–73. doi: 10.1016/j.jmb.2011.09.018 21959263

[39] Wojciechowicz K Spodzieja M Wardowska A . The BTLA-HVEM complex - The future of cancer immunotherapy. Eur J Med Chem. (2024) 268:116231. doi: 10.1016/j.ejmech.2024.116231 38387336

[40] Ye H Park YC Kreishman M Kieff E Wu H . The structural basis for the recognition of diverse receptor sequences by TRAF2. Mol Cell. (1999) 4:321–30. doi: 10.1016/s1097-2765(00)80334-2 10518213

[41] Bjordahl RL Gapin L Marrack P Refaeli Y . iNKT cells suppress the CD8+ T cell response to a murine Burkitt’s-like B cell lymphoma. PloS One. (2012) 7:e42635. doi: 10.1371/journal.pone.0042635 22880059 PMC3413636

[42] Refaeli Y Young RM Turner BC Duda J Field KA Bishop JM . The B cell antigen receptor and overexpression of MYC can cooperate in the genesis of B cell lymphomas. PloS Biol. (2008) 6:e152. doi: 10.1371/journal.pbio.0060152 18578569 PMC2435152

[43] Hurchla MA Sedy JR Murphy KM . Unexpected role of B and T lymphocyte attenuator in sustaining cell survival during chronic allostimulation. J Immunol. (2007) 178:6073–82. doi: 10.4049/jimmunol.178.10.6073 17475832

[44] Albring JC Sandau MM Rapaport AS Edelson BT Satpathy A Mashayekhi M . Targeting of B and T lymphocyte associated (BTLA) prevents graft-versus-host disease without global immunosuppression. J Exp Med. (2010) 207:2551–9. doi: 10.1084/jem.20102017 21078889 PMC2989771

[45] Levental I Grzybek M Simons K . Greasing their way: lipid modifications determine protein association with membrane rafts. Biochemistry. (2010) 49:6305–16. doi: 10.1021/bi100882y 20583817

[46] Stienne C Virgen-Slane R Elmén L Veny M Huang S Nguyen J . Btla signaling in conventional and regulatory lymphocytes coordinately tempers humoral immunity in the intestinal mucosa. Cell Rep. (2022) 38:110553. doi: 10.1016/j.celrep.2022.110553 35320716 PMC9032671

[47] Long EO Kim HS Liu D Peterson ME Rajagopalan S . Controlling natural killer cell responses: integration of signals for activation and inhibition. Annu Rev Immunol. (2013) 31:227–58. doi: 10.1146/annurev-immunol-020711-075005 23516982 PMC3868343

[48] Chen X Trivedi PP Ge B Krzewski K Strominger JL . Many NK cell receptors activate ERK2 and JNK1 to trigger microtubule organizing center and granule polarization and cytotoxicity. Proc Natl Acad Sci. (2007) 104:6329–34. doi: 10.1073/pnas.0611655104 17395718 PMC1851054

[49] Hoek KS Schlegel NC Eichhoff OM Widmer DS Praetorius C Einarsson SO . Novel MITF targets identified using a two-step DNA microarray strategy. Pigment Cell Melanoma Res. (2008) 21:665–76. doi: 10.1111/j.1755-148X.2008.00505.x 19067971

[50] Newman AM Liu CL Green MR Gentles AJ Feng W Xu Y . Robust enumeration of cell subsets from tissue expression profiles. Nat Methods. (2015) 12:453–7. doi: 10.1038/nmeth.3337 25822800 PMC4739640

[51] Pastore A Jurinovic V Kridel R Hoster E Staiger AM Szczepanowski M . Integration of gene mutations in risk prognostication for patients receiving first-line immunochemotherapy for follicular lymphoma: a retrospective analysis of a prospective clinical trial and validation in a population-based registry. Lancet Oncol. (2015) 16:1111–22. doi: 10.1016/S1470-2045(15)00169-2 26256760

[52] Han G Deng Q Marques-Piubelli ML Dai E Dang M Ma MCJ . Follicular lymphoma microenvironment characteristics associated with tumor cell mutations and MHC class II expression. Blood Cancer Discov. (2022) 3:428–43. doi: 10.1158/2643-3230.BCD-21-0075 35687817 PMC9894575

[53] Chen L Flies DB . Molecular mechanisms of T cell co-stimulation and co-inhibition. Nat Rev Immunol. (2013) 13:227–42. doi: 10.1038/nri3405 23470321 PMC3786574

[54] Wu TH Zhen Y Zeng C Yi HF Zhao Y . B and T lymphocyte attenuator interacts with CD3zeta and inhibits tyrosine phosphorylation of TCRzeta complex during T-cell activation. Immunol Cell Biol. (2007) 85:590–5. doi: 10.1038/sj.icb.7100087 17607320

[55] Xu X Hou B Fulzele A Masubuchi T Zhao Y Wu Z . PD-1 and BTLA regulate T cell signaling differentially and only partially through SHP1 and SHP2. J Cell Biol. (2020) 219:e201905085. doi: 10.1083/jcb.201905085 32437509 PMC7265324

[56] Celis-Gutierrez J Blattmann P Zhai Y Jarmuzynski N Ruminski K Grégoire C . Quantitative interactomics in primary T cells provides a rationale for concomitant PD-1 and BTLA coinhibitor blockade in cancer immunotherapy. Cell Rep. (2019) 27:3315–3330.e7. doi: 10.1016/j.celrep.2019.05.041 31189114 PMC6581740

[57] Arifin MZ Leitner J Egan D Waidhofer-Söllner P Kolch W Zhernovkov V . BTLA and PD-1 signals attenuate TCR-mediated transcriptomic changes. iScience. (2024) 27:110253. doi: 10.1016/j.isci.2024.110253 39021788 PMC11253514

[58] Carreras J Lopez-Guillermo A Kikuti YY Itoh J Masashi M Ikoma H . High TNFRSF14 and low BTLA are associated with poor prognosis in Follicular Lymphoma and in Diffuse Large B-cell Lymphoma transformation. J Clin Exp Hematop. (2019) 59:1–16. doi: 10.3960/jslrt.19003 30918139 PMC6528140

[59] Mintz MA Felce JH Chou MY Mayya V Xu Y Shui JW . The HVEM-BTLA axis restrains T cell help to germinal center B cells and functions as a cell-extrinsic suppressor in lymphomagenesis. Immunity. (2019) 51:310–323.e7. doi: 10.1016/j.immuni.2019.05.022 31204070 PMC6703922

[60] Chabot S Jabrane-Ferrat N Bigot K Tabiasco J Provost A Golzio M . A novel antiangiogenic and vascular normalization therapy targeted against human CD160 receptor. J Exp Med. (2011) 208:973–86. doi: 10.1084/jem.20100810 21482699 PMC3092350

[61] Piotrowska M Spodzieja M Kuncewicz K Rodziewicz-Motowidło S Orlikowska M . CD160 protein as a new therapeutic target in a battle against autoimmune, infectious and lifestyle diseases. Analysis of the structure, interactions and functions. Eur J Med Chem. (2021) 224:113694. doi: 10.1016/j.ejmech.2021.113694 34273660

[62] Ward-Kavanagh LK Lin WW Šedý JR Ware CF . The TNF receptor superfamily in co-stimulating and co-inhibitory responses. Immunity. (2016) 44:1005–19. doi: 10.1016/j.immuni.2016.04.019 27192566 PMC4882112

[63] White D Cote-Martin A Bleck M Garaffa N Shaaban A Wu H . Programmed cell death-1 (PD-1) anchoring to the GPI-linked co-receptor CD48 reveals a novel mechanism to modulate PD-1-dependent inhibition of human T cells. Mol Immunol. (2023) 156:31–8. doi: 10.1016/j.molimm.2023.02.007 36889184

[64] Sordo-Bahamonde C Lorenzo-Herrero S Granda-Díaz R Martínez-Pérez A Aguilar-García C Rodrigo JP . Beyond the anti-PD-1/PD-L1 era: promising role of the BTLA/HVEM axis as a future target for cancer immunotherapy. Mol Cancer. (2023) 22:142. doi: 10.1186/s12943-023-01845-4 37649037 PMC10466776

[65] Cheng Y Wang J Yu Y Wang Q Yang R Xia B . Phase I/II study of Tifcemalimab, an anti-B- and T-lymphocyte attenuator antibody, in combination with Toripalimab in previously treated advanced lung cancer. Clin Cancer Res. (2025) 31:2926–34. doi: 10.1158/1078-0432.CCR-25-0022 40378060

[66] Ding C Zhang Y Xia T Li J Yao W Zhang Q . Perioperative the BTLA inhibitor (tifcemalimab) combined with toripalimab and chemotherapy for resectable locally advanced thoracic esophageal squamous cell carcinoma trial (BT-NICE trial): a prospective, single-arm, exploratory study. Front Immunol. (2025) 16:1542877. doi: 10.3389/fimmu.2025.1542877 40276504 PMC12018478

[67] Song Y Ma J Zhang H Xie Y Peng Z Shuang Y . Tifcemalimab as monotherapy or in combination with toripalimab in patients with relapsed/refractory lymphoma: a Phase I trial. Nat Commun. (2025) 16:4559. doi: 10.1038/s41467-025-59461-3 40379637 PMC12084519

[68] Overwijk WW Restifo NP . B16 as a mouse model for human melanoma. Curr Protoc Immunol. (2001) Chapter 20:Unit 20.1. doi: 10.1002/0471142735.im2001s39 18432774 PMC2763508

[69] Schneider CA Rasband WS Eliceiri KW . NIH Image to ImageJ: 25 years of image analysis. Nat Methods. (2012) 9:671–5. doi: 10.1038/nmeth.2089 22930834 PMC5554542

[70] Lenz G Wright GW Emre NCT Kohlhammer H Dave SS Davis RE . Molecular subtypes of diffuse large B-cell lymphoma arise by distinct genetic pathways. Proc Natl Acad Sci USA. (2008) 105:13520–5. doi: 10.1073/pnas.0804295105 18765795 PMC2533222

[71] Dave SS Fu K Wright GW Lam LT Kluin P Boerma EJ . Molecular diagnosis of Burkitt’s lymphoma. N Engl J Med. (2006) 354:2431–42. doi: 10.1056/NEJMoa055759 16760443

