# Supplementary material for: Mutant HVEM in lymphoma disrupts BTLA and CD160 regulation of anti-tumor signaling
Source: Front Immunol. 2026 Sep 11;17:1914960. doi: 10.3389/fimmu.2026.1914960 (PMC13612297; doi:10.3389/fimmu.2026.1914960)
Supplement: Supplementary file 1 [file DataSheet1.pdf]

Supplemental Information

Supplemental Table 1. Primers used for site directed mutagenesis and *Hvem* CRISPR

Gene	Primer	Sequence
Human *HVEM*		
P59S Mutant	hHVEM_P59STop	CAAGTGCAGTTCAGGTTATCGTGTGAAGGAG
P59S Mutant	hHVEM_P59SBot	CGATAACCTGAACTGCACTTGGGGCAGCAC
G60D Mutant	HuHVEMG60Dfor	TGCAGTCCAGATTATCGTGTGAAGGAGGCCTG
G60D Mutant	HuHVEMG60Drev	ACACGATAATCTGGACTGCACTTGGGGC
Y61C Mutant	HuHVEMY61Cfor	GTCCAGGTTGTCGTGTGAAGGAGGCCTGC
Y61C Mutant	HuHVEMY61Crev	CTTCACACGACAACCTGGACTGCACTTGGG
G72D Mutant	hHVEM_G72DTop	GCTGACGGACACAGTGTGTGAACCCTGC
G72D Mutant	hHVEM_G72DBot	ACACACTGTGTCCGTCAGCTCCCCGCAG
T82P Mutant	hHVEM_T82PTop	TCCAGGCCCCTACATTGCCCACCTCAATG
T82P Mutant	hHVEM_T82PBot	AATGTAGGGGCCTGGAGGGCAGGGTTC
Ins91I Mutant	hHVEM_ins91ITop	CTCAATGGCCTAATAAGCAAGTGTCTGCAGTGC
Ins91I Mutant	hHVEM_ins91IBot	CACTTGCTTATTAGGCCATTGAGGTGGGCAATG
A102P Mutant	hHVEM_A102PTop	GTGACCCACCCATGGGCCTGCGCGCG
A102P Mutant	hHVEM_A102PBot	GGCCCATGGGTGGGTCACACATTTGGCACTG
R109W Mutant	HuHVEMR109Wfor	CGCGAGCTGGAACTGCTCCAGGACAGAG
R109W Mutant	HuHVEMR109Wrev	GAGCAGTTCCAGCTCGCGCGCAGGCCC
Q242H Mutant	HuHVEM-Q242H-for	CTCCGTCCATCGGAAAAGACAGGAGGCAGAAG
Q242H Mutant	HuHVEM-Q242H-rev	GTCTTTTCCGATGGACGGAGACGATCACCTTG
E256Q Mutant	HuHVEM-E256Q-for	CACAGTCATTCAGGCCCTGCAGGCCCCTC
E256Q Mutant	HuHVEM-E256Q-rev	CAGGGCCTGAATGACTGTGGCCTCACCTTC
V267M Mutant	HuHVEM-V267M-for	GTCACCACGATGGCCGTGGAGGAGACAATAC
V267M Mutant	HuHVEM-V267M-rev	CACGGCCATCGTGGTGACGTCCGGAGGG
T272I Mutant	HuHVEM-T272I-for	GTGGAGGAGATAATACCCTCATTCACGGGGAG
T272I Mutant	HuHVEM-T272I-rev	TGAGGGTATTATCTCCTCCACGGCCACCG
dCyt Mutant	HuHVEM-dCyt-for	CATATGTGTGTGACCCACAGACTCTGCAC
dCyt Mutant	HuHVEM-dcyt-rev	CTGTGGGTCACACACATATGATTAGGCCAAC
Mouse *Hvem*		
Hvem CRISPR	Hvem-E3-Sg2-ov-FOR	CACCGCATGGGCGGTATATGTCTGT
Hvem CRISPR	Hvem-E3-Sg2-ov-REV	AAACACAGACATATACCGCCCATGC

Supplemental Figure 1

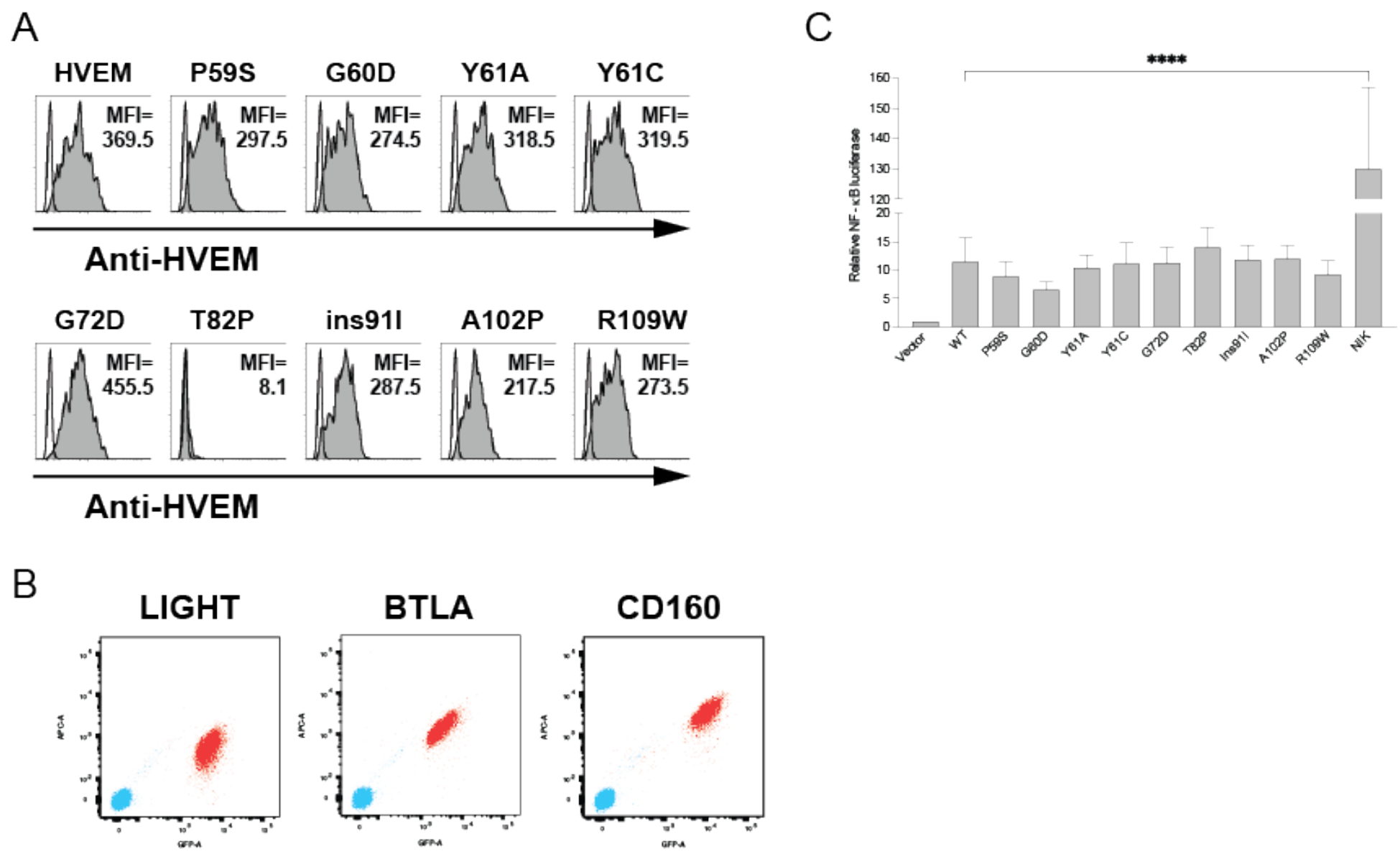

A. 293T cells transduced with wild-type or mutated human HVEM were stained with anti-HVEM. Histograms show HVEM expression (grey) overlaid onto cells transfected with control vector, and the specific MFI is displayed within each plot. **B.** EL4 cells transduced with retroviruses expressing human LIGHT, BTLA and CD160 were analyzed by flow cytometry to confirm ligand expression. **C.** Basal HVEM activation of NF-κB was measured in 293T cells transduced with luciferase reporters and wild-type, lymphoma mutant, or NIK constructs. Graphs show relative NF-κB activation compared to the wild-type HVEM vector control. Data was analyzed by one-way ANOVA with multiple comparisons to the control using the Holm-Šídák test. ****, $p < 0.0001$.

Supplemental Figure 2

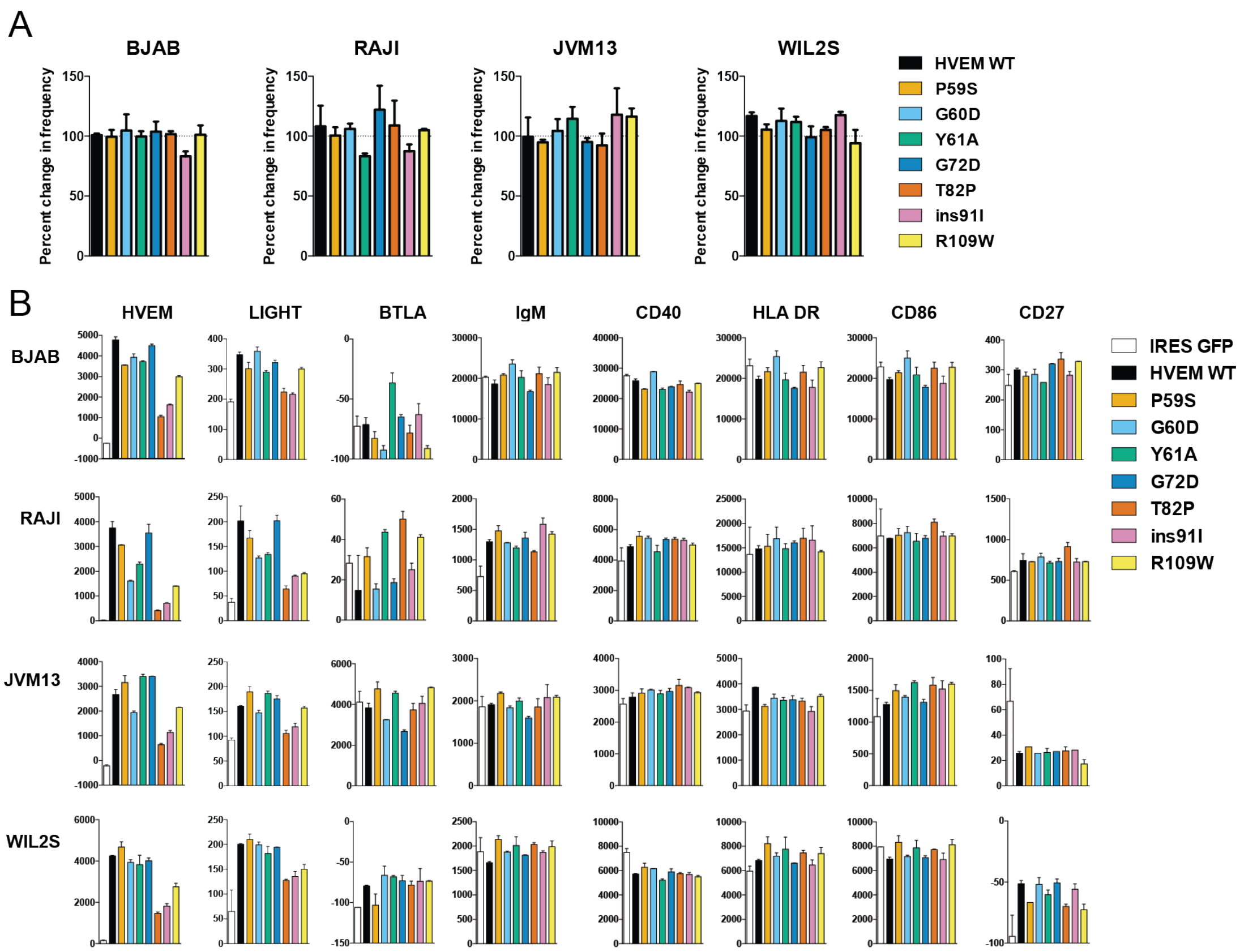

A. BJAB, RAJI, JVM13, and WIL2S lymphoma cell lines transduced with wild-type or mutant HVEM were co-cultured with parental control cells in 4-day proliferation assays. Graphs indicate the percent change in growth of HVEM-expressing cell lines compared to parental controls. Mean ± SEM, representative of two experiments. **B.** The HVEM transduced lymphoma cell lines were analyzed for expression of HVEM, BTLA, LIGHT, IgM, CD40, HLA DR, CD86, and CD27. Graphs in (**B.**) show the MFI of stained cells. Mean ± SEM, representative of two experiments.

Supplemental Figure 3.

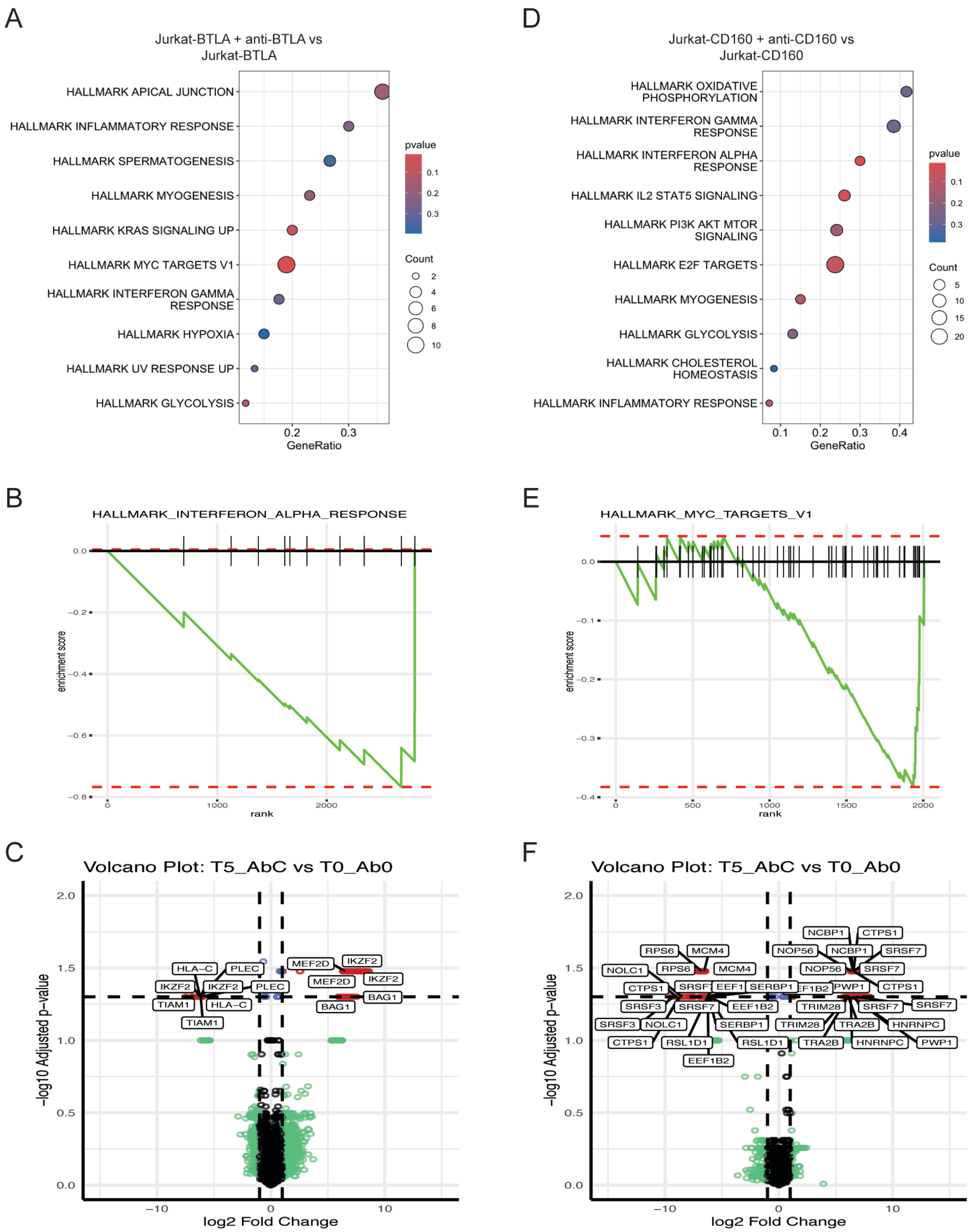

A.-C. Gene set enrichment analysis of differential phospho-protein activation in Jurkat-BTLA cells treated with anti-BTLA compared to cells without treatment. Enriched gene sets are plotted in **A.**, with the INTERFERON_ALPHA_RESPONSE plot in **B**. Volcano plot

of differentially phosphorylated proteins is shown in **C**. **D.-F.** Gene set enrichment analysis of differential phospho-protein activation in Jurkat-CD160 cells treated with anti-CD160 compared to cells without treatment. Enriched gene sets are plotted in **D**., with the MYC_TARGETS_V1 plot in **E**. Volcano plot of differentially phosphorylated proteins is shown in **F**. p values were calculated using the *GSEA* package in R.

Supplemental Figure 4

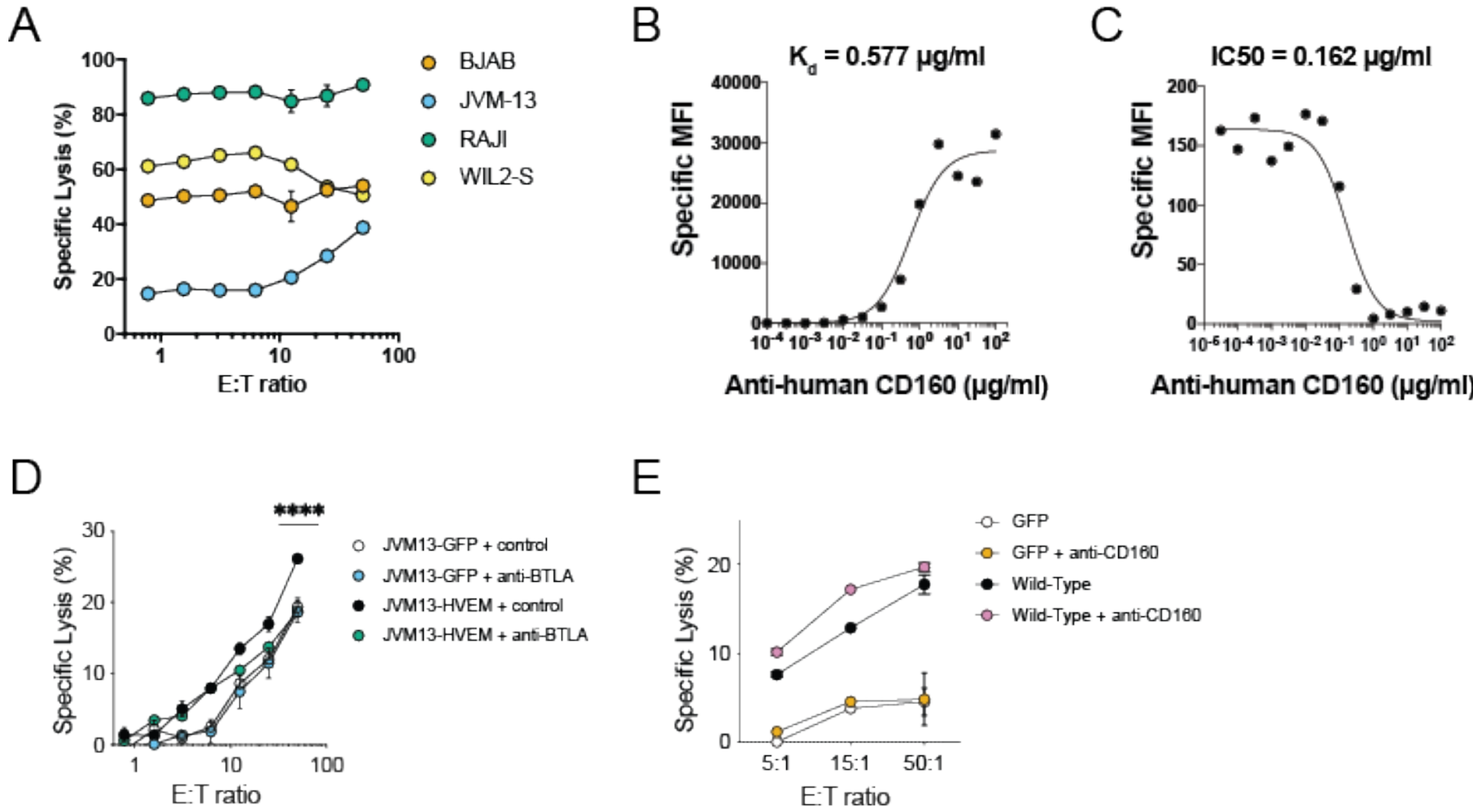

A. NK92 cells were incubated with human lymphoma cell lines at the indicated E:T ratios in cytolysis assays. Graph indicates the specific lysis of different lymphoma cell lines **B.**, **C.** 293T cells transduced with CD160 were incubated with the indicated concentrations of anti-CD160 (clone 688327) alone or prior to staining with 20 µg/ml of HVEM-Fc. Graphs show curves of antibody binding (**B.**) and blockade of HVEM (**C.**). **D.**, **E.** JVM13 cells expressing wild-type HVEM or control vector with the addition of anti-BTLA (**D.**), or anti-CD160 (**E.**) following incubation with effector cells. Data was analyzed by two-way ANOVA with multiple comparisons to the control using the Holm-Šídák test. Mean ± SEM, representative of at least two experiments. ****, $p < 0.0001$.

Supplemental Figure 5

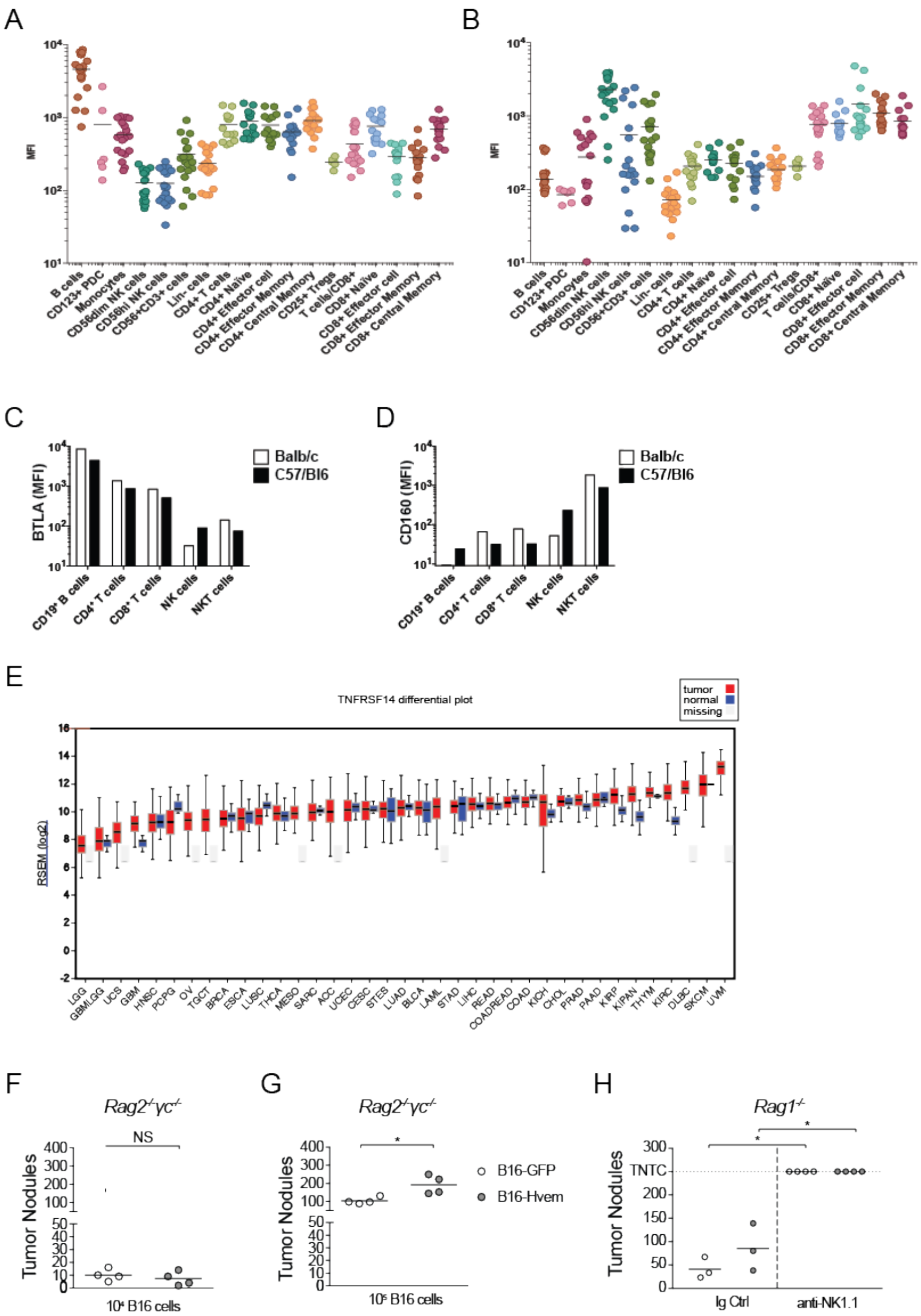

A., **B.** Graph of BTLA expression (**A.**) and CD160 expression (**B.**) (MFI) in human PBMC. **C.**, **D.** Graph of Btla expression (**C.**) and Cd160 expression (**D.**) (MFI) in mouse lymphocyte cell subsets in wild-type Balb/c and C57BL/6 mice. animals. **E**. *TNFRSF14* expression across cancers (Firebrowse.org). **F.-H.** Lungs were harvested from animals 14 days after i.v. inoculation with B16 tumors. *Rag2*$^{-/-}$*Il2rg*$^{-/-}$ recipient animals were inoculated with 10^4 (**F.**) and 10^5 (**G.**) of B16-GFP or B16-HVEM tumor cells. Graphs show numbers

of tumors within lungs at time of harvest. TNTC, too many to count. *Rag1*$^{-/-}$ recipient animals were inoculated with 2.5 × 10^5 B16-GFP or B16-HVEM tumors and were treated with anti-NK1.1 (clone PK136) or isotype control on day 0 and day 2 (**H.**). Data was analyzed using Student's t test. *, $p < 0.05$.

Supplemental Figure 6

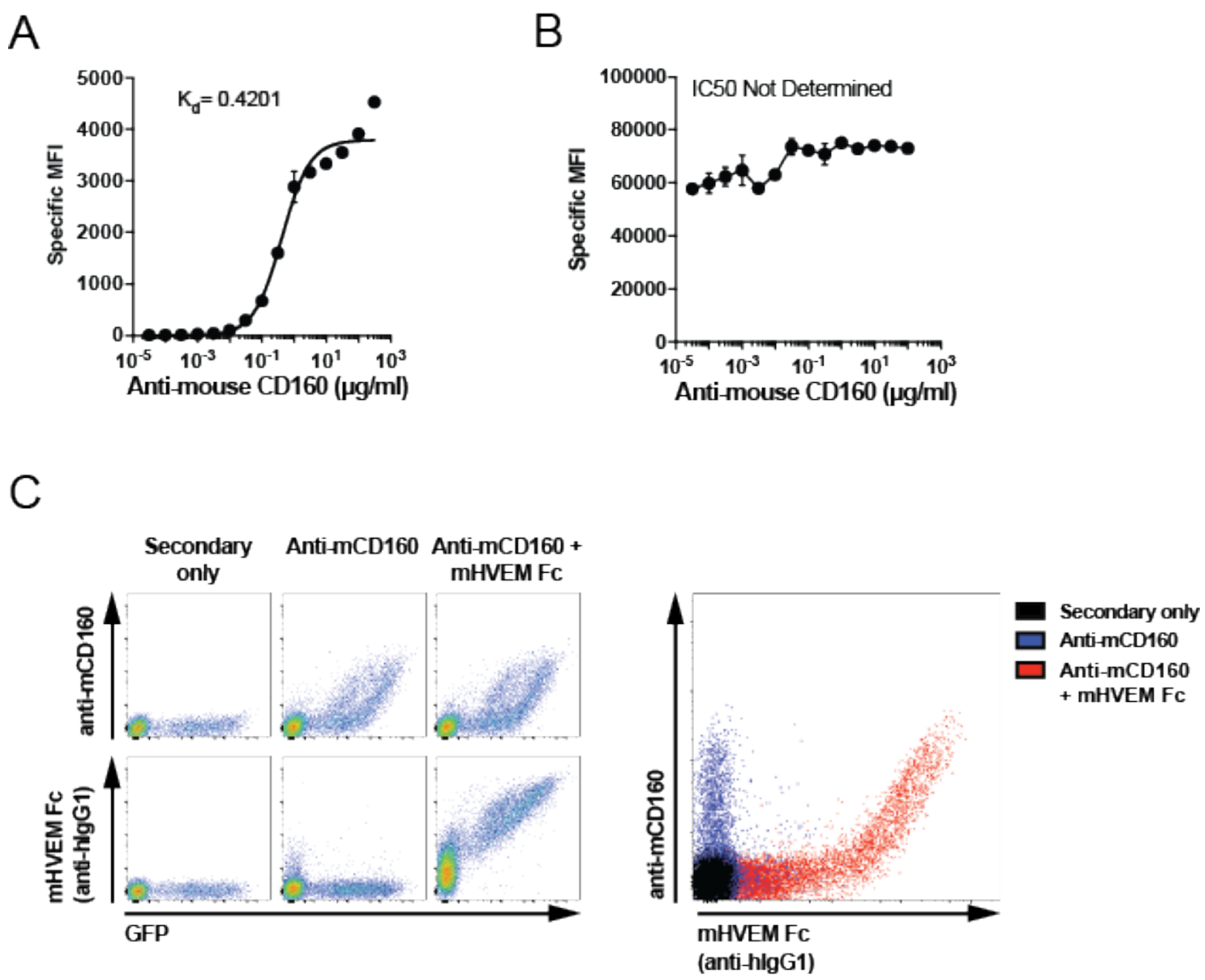

A., **B.** 293T cells transduced with mouse CD160 were incubated with the indicated concentrations of anti-CD160 (clone CNX46-3) alone or with 20 µg/ml mouse HVEM-Fc. Graphs show curves of anti-CD160 binding and calculated K_d (**A.**), and mouse HVEM-Fc binding (**B.**). **C.** Dot plots showing anti-CD160 (top) and mouse HVEM-Fc binding to 293T cells expressing CD160 and control stains. Overlaid plots of anti-CD160 co-binding with mouse HVEM-Fc to 293T cells expressing CD160 and control stains are shown at right.

Supplemental Figure 7

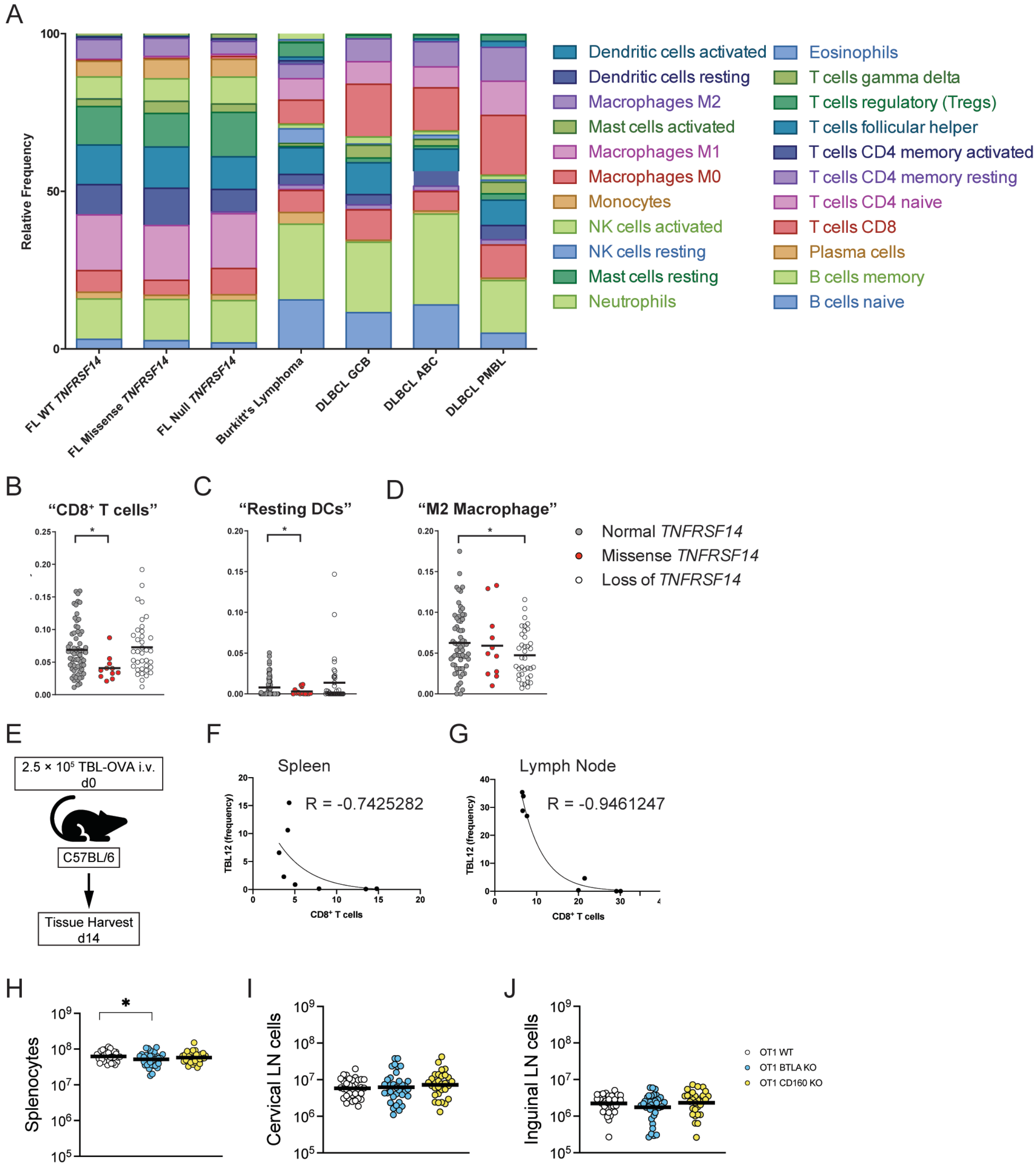

A. Immune signature frequencies calculated using CIBERSORT were analyzed in FL containing either normal, missense, or loss of *TNFRSF14* alleles (GSE66166), Burkitt's Lymphoma (GSE4732), and DLBCL stratified as germinal center- (GCB), activated- (ABC), or primary mediastinal- (PMBL) B cell lymphoma (GSE11318). Loss of *TNFRSF14*

is defined as either a frame shift insertion/deletion, nonsense mutation, splice site mutation, and translational start site mutation. Graph shows the relative frequencies of hematopoietic cell signatures determined using the LM22 dataset. **B.-D.** Graphs show relative frequencies of cell signatures for $CD8^+$ T cells (**B.**), resting dendritic cells (**C.**), and M2 Macrophages (**D.**) in the BCCA validation cohort. **E.** Diagram of experiment for *in vivo* analysis of anti-lymphoma response to TBL cell inoculation in C57BL/6 wild-type animals. **F.**, **G.** Graphs show correlation between frequency of $CD8^+$ T cells and TBL cells in spleen (**F.**) and lymph node (**G.**) with R values indicated. **H.-J.** Graphs show numbers of total splenocytes (**H.**), cervical lymph node cells (**I.**), and inguinal lymph node cells (**J.**) in animals 14 days after inoculation with TBL cells and 7 days after transfer with wild-type, *Btla*$^{-/-}$, and *Cd160*$^{-/-}$ OT1 $CD8^+$ T cells. Data was analyzed by one-way-ANOVA with multiple comparisons using the Holm-Šídák test. * $p < 0.05$, ** $p < 0.01$.
